# Quantitative Proteomics and Transcriptomics Reveals Differences in Proteins During Anthers Development in *Oryza longistaminata*

**DOI:** 10.3389/fpls.2021.744792

**Published:** 2021-11-19

**Authors:** Yue Sun, Xin Wang, Zhongkai Chen, Lu Qin, Bai Li, Linjuan Ouyang, Xiaosong Peng, Haohua He

**Affiliations:** Key Laboratory of Crop Physiology, Ecology and Genetic Breeding, Ministry of Education, Research Center of Super Rice Engineering and Technology, Jiangxi Agricultural University, Nanchang, China

**Keywords:** *Oryza longistaminata*, pollen viability, proteome, fatty acid, glyoxylate cycle, gluconeogenesis

## Abstract

*Oryza longistaminata* is an African wild rice species that possesses special traits for breeding applications. Self-incompatibility is the main cause of sterility in *O. longistaminata*, but here we demonstrated that its pollen vitality are normal. Lipid and carbohydrate metabolism were active throughout pollen development. In this study, we used I_2_-KI staining and TTC staining to investigate pollen viability. Aniline-blue-stained semithin sections were used to investigate important stages of pollen development. Tandem mass tags (TMT)-based quantitative analysis was used to investigate the profiles of proteins related to lipid and carbohydrate metabolism in 4-, 6-, and 8.5-mm *O. longistaminata* spikelets before flowering. Pollen was found to germinate normally *in vitro* and *in vivo*. We documented cytological changes throughout important stages of anther development, including changes in reproductive cells as they formed mature pollen grains through meiosis and mitosis. A total of 31,987 RNA transcripts and 8,753 proteins were identified, and 6,842 of the proteins could be quantified. RNA-seq and proteome association analysis indicated that fatty acids were converted to sucrose after the 6-mm spikelet stage, based on the abundance of most key enzymes of the glyoxylate cycle and gluconeogenesis. The abundance of proteins involved in pollen energy metabolism was further confirmed by combining quantitative real-time PCR with parallel reaction monitoring (PRM) analyses. In conclusion, our study provides novel insights into the pollen viability of *O. longistaminata* at the proteome level, which can be used to improve the efficiency of male parent pollination in hybrid rice breeding applications.

## Introduction

The genus *Oryza* consists of 24 species: two cultivated species (*O. sativa* and *O. glaberrima*) and 22 wild rice species with different ecological adaptations and 10 identifiable genomes (AA, BB, CC, EE, FF, GG, BBCC, CCDD, HHJJ, and HKK; Ge et al., 1999; Zhang et al., 2001). *Oryza longistaminata* (2*n*=24, AA), widely distributed in tropical Africa, is a perennial species with long anthers (Rout et al., 1989), self-incompatibility (Ghesquiere, 1986), strong resistance to biotic and abiotic stresses (Song et al., 1995), and strong rhizomes (Hu et al., 2003). Previous studies have focused mainly on the development of perennial rice (Sacks and Roxas, 2003) and the breeding of blight-resistant varieties, and genes such as Rhz2, Rhz3 (Hu et al., 2003), and Xa21 (Song et al., 1995) have been identified. However, self-incompatibility (Glover et al., 2010) is determined mainly by the recognition of pollen and stigma, and there are few reports on the vitality of the pollen itself. The decoding of the *O. longistaminata* genome is undoubtedly the key to uncovering these significant agronomic characteristics and the molecular mechanisms that enhance rice genetic improvement. However, further study of *O. longistaminata* pollen must be based on cytological analysis (Zhang et al., 2015).

Because the anther development of *O. longistaminata* is completely different from that of *O. sativa*, we must perform new cytological analyses to determine the critical events in its pollen development. The pollen development of *O. sativa* consists of 14 important stages, and the bicellular pollen undergoes its second mitosis during stage 12 (Zhang et al., 2010, 2011). At this stage, the spherical microspores are full of sugar and lipidic materials, among which starch and sucrose provide energy for pollen germination (Feng et al., 2001). In addition, meristem specification, cell differentiation, cell-to-cell communication, meiosis and mitosis are crucial for pollen development (McCormick, 2004; Scott et al., 2004; Ma, 2005; Wilson and Zhang, 2009). Pollen development goes through four important stages, including two meiosis and two mitosis. Pollen development is initiated by the formation of pollen mother cells (PMC) through two rounds of meiosis, leading then to four individual haploid microspores (Ischebeck, 2016; Begcy and Dresselhaus, 2017). The first mitosis represents a key switch in gametophyte formation, defining the transition from microspore development to maturity. During this process, vacuoles begin to decrease in size and number, and mitochondria, plastids, endoplasmic reticulum, and liposomes begin to form gradually (Datta et al., 2002). The Osaocs12 mutant had no mature pollen, mainly because fatty acyl-CoA synthetase OsACOS12 was inhibited at stage 10 (Yang et al., 2017). The *Cut1* gene encodes a long chain fatty acid condensing enzyme that converts fatty acids into cuticular wax (Millar et al., 1999), and many polysaccharide and fatty acid metabolic synthases are necessary for pollen viability and germination. Transcriptome analysis revealed dynamic changes in gene expression between early lipid metabolism and late carbohydrate metabolism during anther development (Li et al., 2016). The second mitosis occurs in rice with three-nucleated pollen (Tanaka, 1997). At this stage, starch filling is completed by entering the pollen grain and the pollen cytoplasm is filled with larger starch grains (Datta et al., 2002). *RIP1* gene affected starch granule formation in rice (Han et al., 2006). The successful completion of pollen germination and pollen tube elongation is also an important factor to measure the maturity of pollen grains and pollen viability. For successful reproduction in higher plants, pollen should germinate only after reaching a compatible stigma and finally growing into the embryo sac within the ovule through the style (Lobstein et al., 2004). *GSL1* and *GSL5* gene were found to affect pollen germination and pollen tube growth in *Arabidopsis* (Guan et al., 2008). In addition, during first mitosis stage, the pollen cytoplasm undergoes several important metabolic changes, including sugar and lipid biosynthesis, protein synthesis and glyoxylate cycle (Niewiadomski et al., 2005).

More importantly, fatty acids undergo β-oxidation for breakdown into acetyl-CoA, which is transformed into citric acid and enters the TCA cycle, where glyoxylate is present. Oxaloacetic acid then produces glucose through gluconeogenesis, thereby achieving the conversion of lipids to sugars. The role of the glyoxylate cycle in the lipid metabolism of seedlings was first discovered in oil crops (Beevers, 1979). Peroxide isomerase (ACN1) converts fatty acids into acetyl-coenzyme A (acetyl-CoA), marking an important entry point for fatty acids into the TCA (Lin and Oliver, 2008). On the other hand, gluconeogenesis may play an important role in glucose metabolism during pollen development (Nakamura et al., 1992). The role of gluconeogenesis in pollen viability and pollen tube germination has also been demonstrated in pine pollen (Malik et al., 1978).

Proteomics is a powerful tool to study protein dynamics and its complex regulatory mechanisms. Since mRNA expression cannot fully reflect protein expression, proteomic analysis has gradually become a useful and widely used method to elucidate cell function (Nobori et al., 2020; Wang et al., 2020). Currently, public databases contain only genome and transcriptome information for *O. longistaminata*. But an understanding of proteins is equally important, particularly those that play a role in pollen development, as proteins are the direct products of gene function and participate directly in pollen metabolism. Here, we performed proteome analyses of staged pollen from *O. longistaminata* and revealed variations in protein expression during the formation of viable pollen. Tandem mass tags-mass spectrometry/mass spectrometry (TMT-MS/MS) analysis was used to identify and quantify low-abundance proteins with high accuracy. We used TMT-MS/MS to track changes in the abundance of proteins involved in energy metabolism during the development of *O. longistaminata* pollen, focusing in particular on proteins related to the biosynthetic pathways of the glyoxylate cycle and gluconeogenesis. Finally, we used real-time PCR and parallel reaction monitoring (PRM) to verify the expression patterns of key genes in the metabolic pathway.

## Materials and Methods

### Plant Materials and Growth Conditions

**Figure 1:**
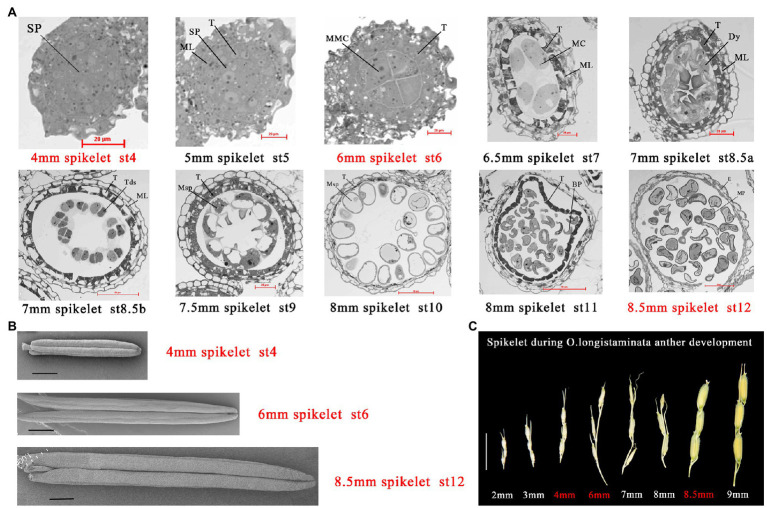
Characterization of male fertility in *Oryza longistaminata*. **(A)** Semi-thin sections of anthers from *O. longistaminata* at different developmental stages. Bars=20 or 50μm. Sp, sporogenous cell; T, tapetum; Tds, tetrads; Dy, dyad cell; E, epidermis; MC, meiotic cell; ML, middle layer; MMC, microspore mother cell; MP, mature pollen; Msp, microspore parietal cell; BP, bicellular pollen; Bars=20 or 50μm **(B)** Scanning electron microscope observation of anther at different developmental stages in *O. longistaminata*. Bars=1 mm. **(C)** Panicle phenotypes of *O. longistaminata* at different developmental stages. Bars=1cm.

*Oryza longistaminata* was introduced from African cultivars of wild rice planted in Hainan Province, China. As *O. longistaminata* is a short-day plant, it was planted in the test base of Jiangxi Agricultural University (18°33'68''N, 109°68'90''E). At Stage 4, archesporial cells undergo periclinal divisions and generate primary sporogenous cells, this stage is the initiation of pollen development (Nonomura et al., 2003). At Stage 6, microspore mother cells (MMCs) begin to form and undergo meiosis, which plays a key role in the formation of pollen fertility. More importantly, the inner secondary parietal layer develops into the tapetum, which means that the anther begins to provide energy for pollen development (Jung et al., 2005). At Stage 12, the most important event is the accumulation of sugar and starch, and the spherical microspores full of reserve substances, which plays a key role in the formation of pollen vitality (Zhang et al., 2010; Figures 1A,B). *Oryza longistaminata* plants were sampled at three important stages of pollen development: 4-mm spikelet (Ls-4mm, primary sporogenous cells, Stage 4), 6-mm spikelet (Ls-6mm, MMCs, Stage 6), and 8.5-mm spikelet (Ls-8.5mm, second mitosis cells, Stage 12; Figure 1C).

### Phenotype Observations and Determination of Pollen Viability

Phenotype analysis included observations of plant type and panicle type, scanning electron microscopy (SEM) observations, I_2_-KI pollen staining, *in vitro* pollen germination tests, observation of pollen tube growth on stigmas, and pollen tube imprinting in the embryo. Pollen (1day before flowering) was stained with 1% I_2_-KI or 2% triphenyltetrazolium chloride (TTC), then observed in three fields to ensure that more than 200 pollen grains were counted for pollen vitality assessment. Sucrose is the main form for storing, accumulating and transporting sugar in plants. It is more effective to cultivate pollen with sucrose than other polysaccharides, so it is the most suitable medium for pollen germination and pollen tube growth. If the sucrose concentration in the medium is too low and the basic nutrients for pollen germination are not enough, pollen germination will be inhibited (Yin and Zhao, 2005). Sucrose concentration of pollen germination *in vitro* of most plants is between 10 and 30%. The mature pollen was shaken into a culture dish that contained *in vitro* germination solution (20, 30, and 40% sucrose, 10% PEG4000, 3mM Ca(NO_3_)_2_, 40mg/L H_3_BO_3_, 3mg/L vitamin B_1_). The experiment was repeated three times, with more than 200 pollen grains counted per replicate. A slide containing stained cells was photographed with an Nikon SMZ1270i stereomicroscope. Six hours after flowering, the stigma was fixed in 75% FAA for 24h and dehydrated in a gradient of 95, 75, 50, 30, and 10% ethanol. The tissue was softened in 2M NaOH for 12h and stained with 0.05% aniline blue for 12h. Then the softened stigma was placed on a clean slide with one drop of glycerin and photographed under a fluorescence microscope. A slide containing stained cells was photographed with an Nikon ECLIPSE Ni fluorescence microscope and Nikon Ds-Ri2 camera system. Pollen tubes were stained with acetocarmine aniline blue, and embryos were stained with 20g/ml H33324. The samples were placed in a mixture of anhydrous ethanol and methyl salicylate (1:1) for 1h, then treated with methyl salicylate twice for 2h each time. The elongation of pollen tubes in mature embryos was observed by confocal laser scanning microscope (WCLSM; Zhao et al., 2013).

### Cytological Observations of Pollen With the Electron Microscope

Samples were fixed in 2% OsO_4_ overnight at 4°C, dehydrated in an ethanol gradient (95, 75, 50, 30, and 10%), and soaked in ethanol-isoamyl acetic acid (V:V=1:3) for 1h. After vacuum coating, the sample was observed under a scanning electron microscope (HITACHI SU8100), and pollen and anthers were photographed. *Oryza longistaminata* anthers at three developmental stages were fixed in 2.5% glutaraldehyde and stored at 4°C. Samples were dehydrated in a PBS gradient (95, 75, 50, 30, and 10%), then embedded in Technovit 7100 resin (Heraeus Kulzer). Microtome sections (5μm) were treated with 0.25% aniline blue to stain the cells. Semi-thin sections of pollen at different developmental stages were photographed with a HITACHI HT7700 transmission electron microscope.

### Determination of Sucrose and Fatty Acid Components

Sugar components were determined by high performance liquid chromatography (HPLC). Anther tissues of *O. longistaminata* were obtained at three stages and dried to constant weight. Dried samples (0.1g) were weighed and ground, and 4ml of ~80% ethanol was added. Samples were placed in a water bath at 80°C, stirred every 5min for a total of 40min, and the supernatant was collected after centrifugation. The extraction was repeated three times, and the supernatants were collected in a test tube; then 20mg of activated carbon was added to the tube, and the liquid was vibrated and decolorized for 30min in a water bath at 80°C, with vibration once every 10min. Then the filtrate was filtered twice, poured into an evaporating dish, evaporated in a water bath at 80°C, and then dissolved in 0.5ml ultra-pure water. The solution was filtered through a 0.2-μm water system membrane. Finally, the sugar extract was placed in a 1.5-ml centrifuge tube for the determination of sucrose content by HPLC. The chromatographic conditions were: Agilent 1100 HPLC system, differential refractive detector, and NH_2_ analysis column. The flow rate of mobile phase was 1.0ml/min, the column temperature was 30°C, and the sample volume was 20μl. The optimum chromatographic conditions (acetonitrile/water=80:20, *v*/*v*) were selected for analysis. The peak area of sucrose was measured and recorded, and the content of sucrose at different developmental stages was calculated. Three biological replicates were performed for each sample type.

Fatty acid components were determined by gas chromatography–mass spectrometry (GC-MS; Sanchez-Salcedo et al., 2016). Anther tissues of *O. longistaminata* were obtained from three developmental stages. Each anther sample (0.25g) was weighed and placed in a 5-ml volumetric flask; 1ml 0.5M KOH-methyl alcohol was added, and the mixture was heated at 65°C for 30min. At the end of alkalolysis, 1ml of 12.5% BF_3_-methanol was added, and the mixture was heated at 65°C for 30min for esterification. After esterification, 1ml paraffin ether was added to extract the fatty acid methyl esters by full oscillation, and the ester was left standing. Then 1ml saturated NaCl was slowly added to the volumetric flask, and the upper organic phase containing fatty acid methyl esters was used for the detection of fatty acid composition by gas chromatography. In this system, the flow rate of helium was 20ml/min, and the inlet and transfer line temperatures were 210 and 200°C, respectively. The heating procedure was optimized as follows: from 40 to 140°C at a rate of 20°C/min, increasing to 180°C at a rate of 4°C/min, and up to 200°C for 5min. Fatty acid composition was determined based on peak time and comparison with the standard fatty acid methyl ester. The area percentage of each component was taken as its percentage content.

### RNA Sequencing

Total RNA was extracted from pollen of three developmental stages by grinding tissue using the TRIzol reagent (Invitrogen). DNase digestion was performed to avoid contamination from genomic DNA, and the phenol chloroform method was used to isolate the total RNA. The integrity of the extracted RNA was measured with a NanoDrop 2000 spectrophotometer (Thermo Scientific) based on the 260/280 and 260/230nm absorbance ratios. mRNA was enriched using Oligo(dT) magnetic beads, then sheared into short fragments with fragmentation buffer. The mRNA was used as a template to synthesize first-strand cDNA with random hexamer primers, and the second-strand cDNA was synthesized by adding buffer, dNTPs, RNase H, and DNA polymerase I. After purification with the QiaQuick PCR kit and elution with EB buffer, end repair was performed, poly(A) was added, and sequencing ligators were attached. Fragment size selection was performed by agarose gel electrophoresis, and then PCR amplification was performed. The constructed sequencing libraries were sequenced on the Illumina Hiseq 2000 platform.

Raw image data generated by the sequencer were converted to raw reads by base calling, and the results were stored in FastQ file format. Raw data (raw reads) of fastq format were firstly processed through in-house perl scripts. In this step, clean data (clean reads) were obtained by removing reads containing adapter, reads containing ploy-N and low-quality reads from raw data. At the same time, 20, Q30, and GC content the clean data were calculated. HiSat2 software was used to compare clean reads and obtain unique reads (Kim et al., 2015). All the downstream analyses were based on the clean data with high quality. The distribution and coverage of unique reads on the reference sequence^1^ were counted to determine whether the comparison results passed quality control test and to confirm that there were sufficient high-quality reads for subsequent bioinformatics analysis. Reference genome and gene model annotation files were downloaded from genome website directly. Index of the reference genome was built using Hisat2 v2.0.5 and paired-end clean reads were aligned to the reference genome using Hisat2 v2.0.5. We selected Hisat2 as the mapping tool for that Hisat2 can generate a database of splice junctions based on the gene model annotation file. The RNA-seq data used in this study are deposited in NCBI Gene Expression Omnibus database (accession no. GSE181045).

### Protein Extraction and Trypsin Digestion

Anther samples from three developmental stages were thoroughly ground to powder in liquid nitrogen. Samples from each group were combined with a four-fold volume of phenol extraction buffer, then ultrasonically fragmented. An equal volume of Tris was added, and the mixture was centrifuged at 5,500*g* and 4°C for 10min. The supernatant was combined with a five-fold volume of 0.1M ammonium acetate/methanol for precipitation overnight. Protein precipitates were washed with methanol and acetone, then redissolved in 8M urea, and the protein concentration was determined with a BCA kit. The same amount of each protein sample was taken for enzymatic hydrolysis. The final concentration of 20% trichloroacetic was added slowly, then the mixture was vortexed and precipitated at 4°C for 2h. The supernatant was discarded after centrifugation at 4,500*g* for 5min. The pellet was washed and precipitated with pre-cooled acetone 2–3 times. After the precipitate was dried, TEAB was added to a final concentration of 200mM, the precipitate was dispersed by ultrasound, and trypsin was added at a ratio of 1:50 for enzymatic hydrolysis overnight. DTT was added to a final concentration of 5mM and reduced at 56°C for 30min. Finally, IAA was added to a final concentration of 11mM and incubated at room temperature in the dark for 15min.

### LC-MS/MS Quantitative Proteomics

For liquid chromatography, the peptides were dissolved in mobile phase A and separated using a nanoElute ultra-performance liquid system (Bruker). Mobile phase A was an aqueous solution of 0.1% formic acid and 2% acetonitrile. Mobile phase B was a solution of 0.1% formic acid and 100% acetonitrile. The liquid phase gradient settings were: 0–70min, 5–22% B; 70–84min, 22–32% B; 84–87min, 32–80% B; 87–90min, 80% B. The flow rate was maintained at 300nl/min. After separation by the ultra-performance liquid system, the peptide was injected into the capillary ion source for ionization and then analyzed by timsTOF3 mass spectrometry. The voltage of the ion source was set to 2.0kV. High-resolution TOF was used to detect and analyze the parent ions of the peptide and its secondary fragments. The secondary mass spectrum scanning range was set to 100–1,700. Data collection used the parallel cumulative serial fragmentation (PASEF) mode. After one primary mass spectrometry collection, the secondary spectrograms with the charge number of parent ions in the range of 0–5 were collected in PASEF mode 10 times. The dynamic exclusion time of tandem mass spectrometry was set at 30s to avoid repeated scanning of parent ions (Cox et al., 2009). The mass spectrometry proteomics data are available at the ProteomeXchange Consortium *via* the PRIDE partner repository with the dataset identifier PXD028805.

Protein annotation mainly include Kyoto Encyclopedia of Genes and Genomes (KEGG) annotation and subcellular localization. KEGG database was used to annotate protein pathway. Firstly, BLAST comparison (blastp, *e*-value ≤1*e*-4) was performed on the identified proteins. For BLAST comparison results of each sequence, the result with the highest score was selected for annotation. Then using KEGG online service tools KAAS to annotated protein’s KEGG database description. Finally, mapping the annotation result on the KEGG pathway database using KEGG online service tools KEGG mapper. We used WoLF PSORT a subcellular localization predication soft to predict subcellular localization. WoLF PSORT is an updated version of PSORT/PSORT II for the prediction of eukaryotic sequences.

### qRT-PCR Analysis

RNA extraction was performed as described for the RNA-seq procedure. cDNA was synthesized using a PrimeScript 1st strand cDNA synthesis kit (TaKaRa) according to the instructions of the PrimeScript RT Master Mix. Complementary DNA was amplified and quantified using a 7500 Real-Time PCR system (Thermo Scientific) with SYBR Green PCR Master Mix (TaKaRa). Each sample was analyzed in triplicate, and the relative mRNA expression fold changes, calculated with the 2^−ΔΔ*C*T^ method, were normalized to the housekeeping gene *Actin* (gene ID: Os03g0718100). The program was set as follows: holding for 5min at 95°C followed by 40cycles of 15s at 95°C, 15s at 57°C, and 30s at 62°C. qRT-PCR primers were designed using the online NCBI Primer-blast tool (Supplementary Table S7).

### PRM Analysis

Proteins were extracted as described for the proteomic procedure. The peptides were dissolved in liquid chromatography mobile phase A and separated using the EASY-NLC 1000 UPLC-system. Mobile phase A was an aqueous solution of 0.1% formic acid and 2% acetonitrile. Mobile phase B was an aqueous solution of 0.1% formic acid and 90% acetonitrile. The liquid phase gradient settings were: 0–16min, 8–30% B; 16–22min, 30–40% B; 22–26min, 40–80% B; 26–30min, 80% B. The flow rate was maintained at 500nl/min. The peptides were separated with an ultra-performance liquid phase system, injected into an NSI ion source for ionization, and then analyzed by Q Exactive Plus mass spectrometry. The ion source voltage was set at 2.1kV. Orbitrap was used to detect and analyze the peptide parent ions and their secondary fragments. The scanning range of primary mass spectrometry was set to 382–953*m*/*z*, and the scanning resolution was set to 70,000. The Orbitrap scanning resolution was set to 17,500. The data acquisition mode used a data-independent scan (DIA) program, and the HCD collision pool fragmentation energy was set to 27. The automatic gain control (AGC) of primary mass spectrometry was set to 3E6, and the maximum ion implantation time (Maximum IT) was set to 50ms. The AGC of secondary mass spectrometry was set to 1E5, the maximum IT to 220ms, and the isolation window to 1.6*m*/*z*. For peptide parameters, the protease was set to Trypsin [Kr/P], the maximum number of missed cut sites was set to 0, the length of the peptide was set to 7–25 amino acid residues, and cysteine alkylation was set as fixed modification. For transition parameters, the parent ion charge was set to 2, 3; the child ion charge to 1; and the ion type to B, Y. Fragment ion selection began from the third to the last, and the tolerance of mass error for ion matching was set to 0.02Da (Glen et al., 2010). In the experimental design, more than two unique peptides were used for quantitative analysis of each protein (only one unique peptide was suitable for PRM verification for some proteins), and only one peptide was identified for some proteins due to sensitivity and other reasons. After normalizing the quantitative information by the heavy isotope-labeled peptide, a relative quantitative analysis (three biological replications) was performed on the target proteins. The protein relative expression level were processed and analyzed using Microsoft Excel 2007 and SPSS 16.00. Data of qRT-PCR and PRM were analyzed by one-way ANOVA, and treatment means were compared using the least significant difference test (LSD) at the *p*=0.05 probability level. Figures were constructed using Origin 17.0 (Origin Lab, Northampton, MA, United States).

### Subcellular Localization of OlPCP Gene

The coding sequence of OlPCP gene without stop codon was amplified through RT-PCR with 2μl cDNA from panicles in *O. longistaminata*. Then the PCR products was ligated with EcoR I and Kpn I-digested pAN580-eGFP-N1 vector to generate pAN580- OlPCP -GFP fusion expression vector derived by CaMV 35S promoter. The constructed OlPCP -GFP fusion expression vector was confirmed by sequencing. This fusion vector and the control vector (pAN580-eGFP-N1) were transformed into Rice protoplast cells by PEG-mediated method, respectively. Green fluorescent protein (GFP) fluorescence in transformed Rice protoplast cells was detected under a laser scanning confocal microscope.

### Bioinformatics Analysis

The protein/transcriptome data with quantitative values in all samples were selected for dimensionality reduction. The data were first transformed by log2, and the mean value was subtracted. Then the PRCOMP function in R was used for principal component analysis. Gene expression was calculated using an FPKM algorithm (DESeq2). Differentially expressed genes (DEGs) between pairs of developmental stages were identified based on a false discovery rate (FDR) ≤0.001 and |log2Ratio|≥1 (Love et al., 2014). With 4-mM spikelets as a control, the expression of each gene calculated relative to its expression in 4-mm spikelets. The KEGG database was used to identify enriched pathways in DEG sets relative to all identified proteins using a two-tailed Fisher’s exact test. Pathways with FDR-corrected values of *p*<0.05 were considered to be significantly enriched. These pathways were classified into hierarchical categories using the KEGG website. For further hierarchical clustering based on differentially expressed protein functional classifications (such as GO, Domain, Pathway, and Complex), we first collated all the enriched categories along with their values of *p*, then filtered for those categories that were enriched in at least one of the clusters with a value of *p* <0.05. This filtered value of *p* matrix was transformed using the function *x*=−log_10_ (*p*). Finally, these *x* values were *z*-transformed for each functional category. The *z*-scores were then clustered by one-way hierarchical clustering (Euclidean distance, average linkage clustering) in Genesis. Cluster membership was visualized with a heat map using the heatmap.2 function from the gplots R-package.

## Results

### Developmental Stages of *Oryza longistaminata* Pollen

*Oryza longistaminata* generally flowers in succession from January to May in Sanya, China under short-day conditions. Because of differences in pollen development between *O. longistaminata* and *O. sativa*, we examined transverse sections of *O. longistaminata* anthers at multiple developmental stages (Figure 1A). The 4-mm spikelet was in stage 4 of pollen development, in which cell divisions generate the sporogenous primary cells. The 5-mm spikelet was in stage 5, when the primary sporogenous cells divide and form the sporogenous cells, and the presence of the tapetum indicates that the spore mother cell has begun to supply nutrients. The 6-mm spikelet was in stage 6, in which four layers of anther somatic cells enclose the anther locule, where MMCs contact the tapetum. The 6.5-mm spikelet was in stage 7, when meiosis begins. The 7-mm spikelet was in stages 8a and 8b, when one meiocyte forms a dyad cell at the end of meiosis I, and the dyad cell then forms tetrads at the end of meiosis II, and cavitation appears in the tapetum. The 7.5-mm spikelet was in stage 9, when haploid microspores are released, and the middle layer has degraded. The 8-mm spikelet was in stages 10 and 11, when the microspore becomes more vacuolated with a round shape, then forms bicellular pollen, which undergoes the first mitotic division. The 8.5-mm spikelet was in stage 12, when bicellular pollen forms mature pollen. The most important event at this stage is the accumulation of sugar, and mature pollen begins to pick up energy. The study also observed anther morphology at three key stages of pollen development (Figure 1B). In order to properly time transcriptome and proteome sampling, we needed to observe pollen development in the panicle (Figure 1C).

### Confirmation of *Oryza longistaminata* Pollen Viability

**Figure 2:**
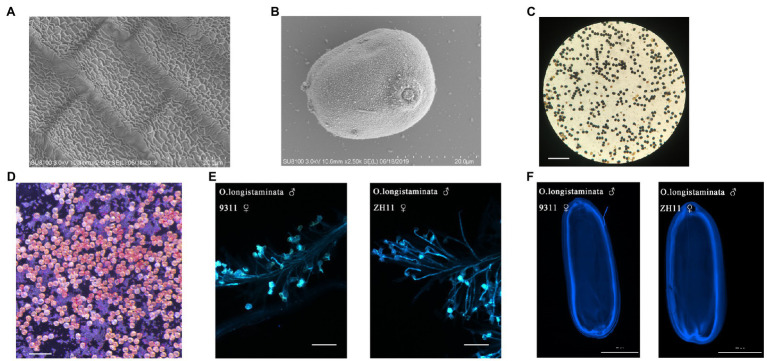
Cytological analysis of pollen development in *O. longistaminata*. **(A)** Scanning electron microscope observation of the anther cuticle in O. longistaminata. Bars=20μm. **(B)** Scanning electron microscope observation of pollen grains in O. longistaminata. Bars=20μm. **(C)** I2-KI staining of pollen. Bars=200μm. **(D)** Triphenyltetrazolium chloride (TTC) staining of pollen. Bars=100μm. **(E)** Pollen tube growth of O. longistaminata on the stigmas of 9311 and ZH11. Bars=100μm. **(F)** The pollen tube imprinting of O. longistaminata on mature embryos of 9311 and ZH11. Bars=2,000μm.

But the panicles do not set seed. To confirm that pollen viability is normal, we observed the mature anther cuticle and pollen grains of *O. longistaminata* using SEM: the mature anthers had obvious reticulate anther cuticles, and the pollen grains of *O. longistaminata* were round (Figures 2A,B). The 8.5-mm spikelet was in stage 12. At this stage, the generative cell in the microspore undergoes the second mitosis and the mature pollen grain contains three nuclei (tricellular pollen). The anther development and pollen maturation are nearly complete (Feng et al., 2001). From stage 12 to flowering, Pollen grains become more spherical in shape. The lemma and palea interlocking structure is opened and the filament elongates. The septum breaks, and the anther continues the release of mature pollen grains. Pollen grains (97.8%, *n*=800) were stained with I_2_-KI, and the results showed completely normal viability of the male gametes (Figure 2C). Pollen grains (96.2%, *n*=630) were stained with TTC, which showed that pollen activity was very strong (Figure 2D). The viability *O. longistaminata* pollen was next evaluated by pollinating indica (9311) and Japonica (ZH11) flowers with *O. longistaminata* pollen grains. In both ZH11 and 9311, pollen tubes grew normally on the stigma 6h after flowering (Figure 2E). After pollination, the pollen grain germinates and the pollen tube passes through the stigma, enters the micropyle, and releases a sperm nucleus which fertilizes the egg cell to form a zygote and develops into an embryo (Chang et al., 2020). The embryo grows gradually within 6days after pollination, and pollen tube staining was retained in the mature embryo 6days after flowering. The pollen tube staining showed that the pollen tubes had reached the position of the ovules in the mature embryo sac (Figure 2F). These results indicate that male fertility is not impaired in *O. longistaminata*.

### Sucrose and Fatty Acid Components During Critical Developmental Stages in *Oryza longistaminata*

**Figure 3:**
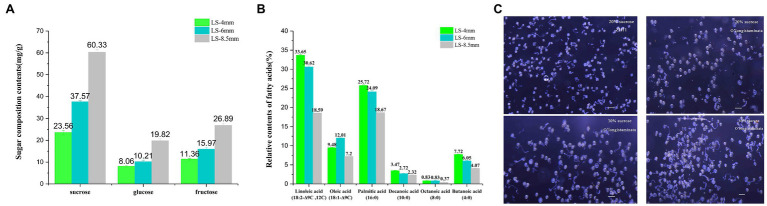
Metabolite analysis of pollen development and an *in vitro* pollen germination assay in *O. longistaminata*. **(A)** Concentrations of three sugar components in pollen from 4-, 6-, and 8.5-mm spikelets. **(B)** Relative contents of different fatty acids in pollen from 4-, 6-, and 8.5-mm spikelets. **(C)** Pollen germination rate at different sucrose concentrations in *O. longistaminata* and *O. sativa*. The sugar composition tests were performed with three replicates, each consisting of 0.1g anther sample. The relative content of fatty acids tests were performed with three replicates, each consisting of 0.25g anther sample. Values are mean±standard error. Data followed by different lower-case letters denote significant differences between sugar composition/relative content of fatty acids at the 5% level according to least significant difference test (LSD) test.

We identified sugar components in pollen extracts from spikelets at three developmental stages using HPLC. The contents of sucrose, glucose, and fructose increased steadily from the 4-mm spikelet to the 8.5-mm spikelet and were all highest in the 8.5-mm spikelet: sucrose 60.33mg/g, glucose 19.28mg/g, and fructose 26.89mg/g (Figure 3A; Supplementary Table S1). Likewise, we identified the relative contents of different fatty acids in pollen extracts from spikelets at three developmental stages using GC-MS. The contents of linoleic acid (18:2^−Δ9C,12C^), oleic acid (18:1^−Δ9C^), palmitic acid (16:0), decanoic acid (10:0), octanoic acid (8:0), and butanoic acid (4:0) declined from the 4-mm spikelet to the 8.5-mm spikelet, and all were highest in the 4-mm spikelet: linoleic acid 33.65%, palmitic acid 25.72%, decanoic acid 3.47%, octanoic acid 0.83%, and butanoic acid 7.72% (Figure 3B; Supplementary Table S2).

Pollen activity was quantified with a pollen germination test, which confirmed the completely normal viability of male gametes from *O. longistaminata* and *O. sativa*. However, in 20% sucrose, the germination rate of *O. longistaminata* (61.3%, *n*=430) was lower than that of ZH11 (83.5%, *n*=380). The germination rate of *O. longistaminata* (85.3%, *n*=420) was better in 40% sucrose solution (Figure 3C), suggesting that *O. longistaminata* pollen contains higher levels of low molecular weight sugars, such as sucrose.

### Transcriptomes and Proteomes at Three Pollen Developmental Stages

**Figure 4:**
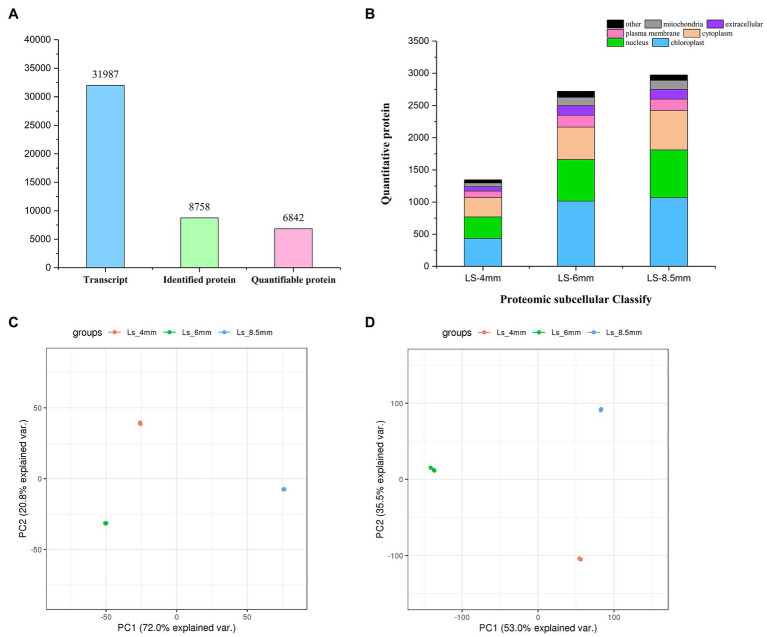
Transcriptomics and proteomics in O. longistaminata pollen. **(A)** Number of mRNAs/proteins detected in the RNA-seq/proteome analysis. **(B)** Number of proteins with different subcellular localizations. **(C)** PCA plot showing the quantitative repeatability of the transcriptome. **(D)** PCA plot showing the quantitative repeatability of the proteome.

We analyzed the transcriptome and proteome of *O. longistaminata* using RNA-sequencing (RNA-seq) and liquid chromatography-mass spectrometry (LC-MS), respectively. Cytology revealed the critical stages of pollen development (4-mm, 6-mm, and 8.5-mm spikelets), and RNA and protein were extracted from isolated anther tissue for RNA-seq and LC-MS analysis, respectively (Supplementary Figure S1A). In total, 31,987 transcripts and 8,758 proteins were detected and identified, and 6,842 of the proteins were quantified (Figure 4A). Proteins showed similar subcellular distributions during the three developmental stages, and chloroplast-related proteins were most abundant (Figure 4B). Correlation cluster heat map showed good repeatability of protein/mRNA data during analysis of the three developmental stages (Supplementary Figures S1B,C). PCA analysis suggested that no obvious biases or batch effects were introduced into the mRNA/protein data during analysis of the three developmental stages. Replicate samples from the same developmental stage clustered together in PCA plots for both the transcriptome and proteome (Figures 4C,D). In both proteomics and transcriptomics, the correlation coefficients of protein and corresponding mRNA were *R*^2^=0.499, 0.184, and 0.368, respectively. The results indicated that in addition to the one-to-one correspondence relationship between transcriptome and proteome, there were other more complex regulatory relationships (Supplementary Figure S2).

**Figure 5:**
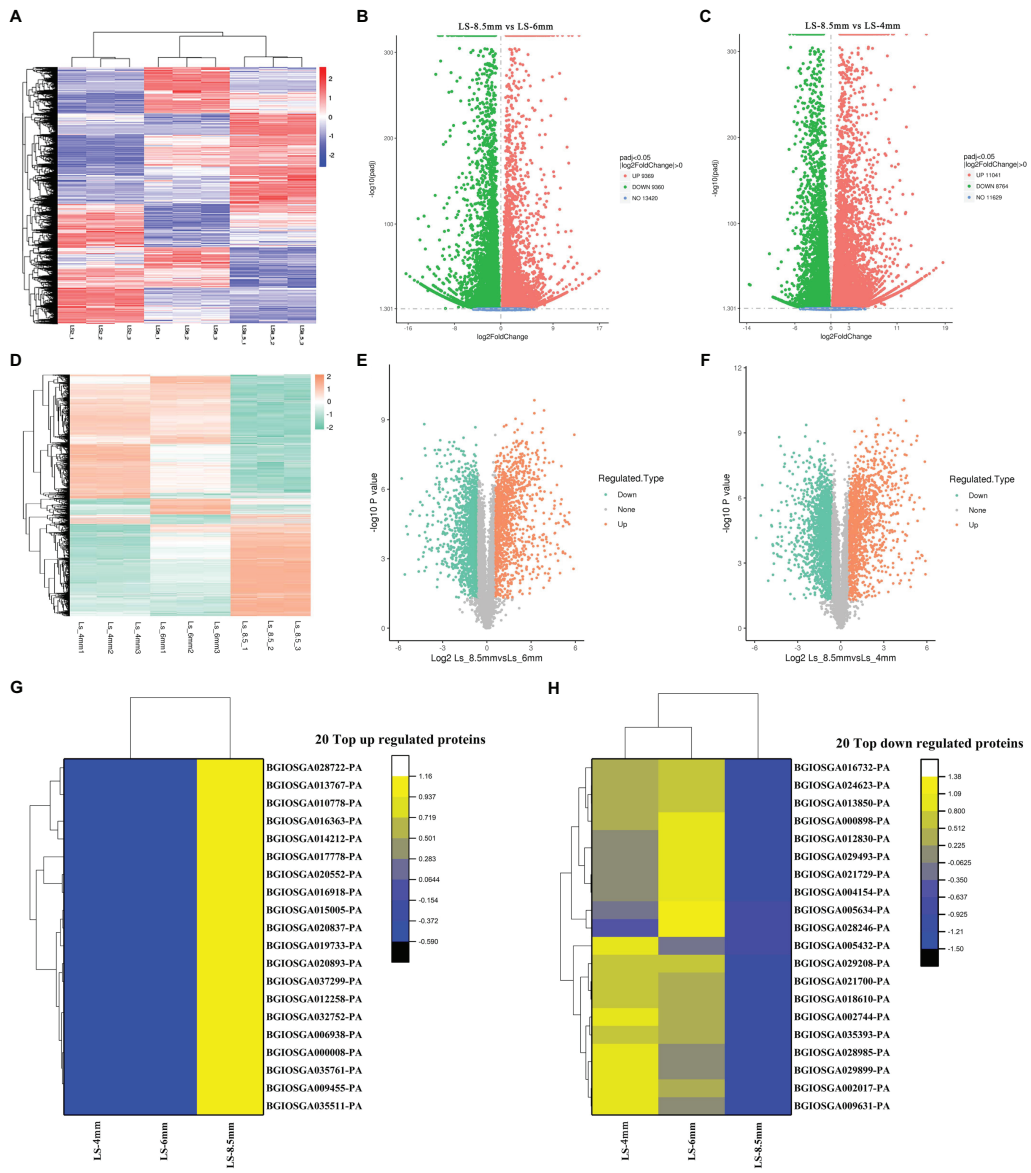
Differentially expressed genes (DEGs) and differentially expressed proteins (DEPs) between anthers from different spikelet growth stages in *O. longistaminata*. **(A)** Heatmap of fold-change differences in gene expression among samples from three spikelet stages. **(B)** Volcano plot of DEGs between Ls-8.5mm and Ls-6mm. **(C)** Volcano plot of DEGs between Ls-8.5mm and Ls-4mm. **(D)** Heatmap of fold-change differences in protein expression among samples from three spikelet stages. **(E)** Volcano plot of DEPs between Ls-8.5mm and Ls-6mm. **(F)** Volcano plot of DEPs between Ls-8.5mm and Ls-4mm. The ordinate represents the multiple of difference and the ordinate represents the level of significance. The scatter in the upper left corner represents significant gene/protein expression, differentially upregulated. The scatter in the upper right represents significant gene/protein expression, differentially upregulated. **(G)** Heat map of 20 top up regulated proteins at Ls-4mm, Ls-6mm and Ls-8.5mm spikelets. **(H)** Heat map of 20 top down regulated proteins at Ls-4mm, Ls-6mm and Ls-8.5mm

Transcriptome and proteome heatmaps showed distinct patterns of gene expression (Figure 5A) and protein abundance (Figure 5D) at different developmental stages. All transcripts and quantified proteins were compared between pairs of pollen developmental stages, and the threshold for differential expression was *p*adj<0.05 and |log2 foldchange|>0. Three clusters corresponding to the three sampled stages were identified for both the transcriptome and proteome. Genes were differentially expressed between Ls-8.5mm and Ls-6mm, of which 9,369 were upregulated in Ls-8.5mm and 9,360 were downregulated (Figure 5B). Genes were differentially expressed between Ls-8.5mm and Ls-4mm, of which 11,041 were upregulated in Ls-8.5mm and 8,769 were downregulated (Figure 5C). Among the quantified proteins, proteins were differentially expressed between Ls-8.5mm and Ls-6mm, of which 1,184 were upregulated in Ls-8.5mm and 1,535 were downregulated (Figure 5E). Proteins were differentially expressed between Ls-8.5mm and Ls-4mm, of which 1,283 were upregulated in Ls-8.5mm and 1,689 were downregulated (Figure 5F). Relative to the 4-mm spikelets, there were more differentially expressed proteins (DEPs) in the 8.5-mm spikelets than in the 6-mm spikelets. In the top left and top right corner of the volcano map are the concentrations of different genes, which are the focus of study. The proteome sequencing results of this study were based on the transcriptome data of this study. Therefore, we paid more attention to the top 20 up and to 20 down regulated proteins from 6mm spikelets to 8.5mm spikelets (Figures 5G,H). Top 20 down- and to 20 upregulated proteins correspond to the top left and top right corner of the volcano map, respectively. There were some upregulated proteins related to pollen development and some downregulated proteins related to carbohydrate synthesis in top20 proteins. Upregulated proteins included probable lipid transfer (*BGIOSGA035511-PA*, 2.00), GDSL-like Lipase/Acylhydrolase (*BGIOSGA014212-PA*, 1.33), GH3 auxin responsive promoter (*BGIOSGA017778-PA*, 2.59), and Ribonuclease T2 family (*BGIOSGA028722-PA*, 2.96). GDSL-like Lipase family genes are a class of lipolytic enzymes (Akoh et al., 2004). Ribonuclease family gene is related to the Tapetal cell layer that surrounds the pollen SAC, which determines pollen fertility (Celestina et al., 1990). On the other hand, the downregulation of cellulose synthase (*BGIOSGA024623-PA*, 0.07) was the most significant, indicating that anther wall stopped growing and burst at late pollen development (Gutierrez et al., 2009; Supplementary Tables S9 and S10).

### Cluster Analysis of mRNAs and Identified Proteins

**Figure 6:**
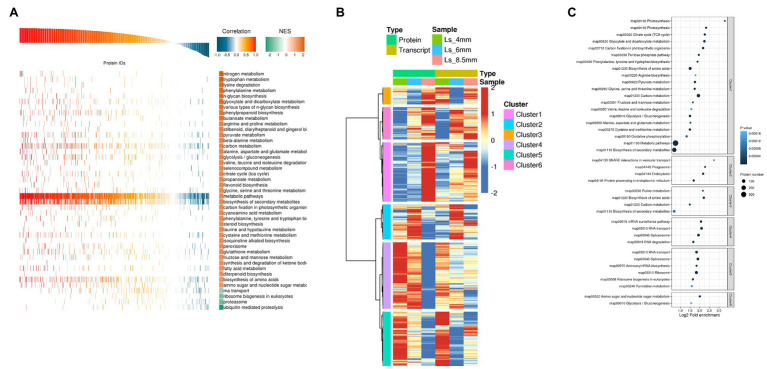
Correlation analysis of transcriptome and proteome data from *O. longistaminata* anthers of different spikelet growth stages. **(A)** Protein and transcript GEST analysis. Pathways with NOM *p*<0.05 were used to draw a GSEA analysis chart. Correlation is close to 1.0, indicating a positive correlation between gene translation and protein translation. Correlation is close to −1.0, indicating a negative correlation. The reliability of Kyoto Encyclopedia of Genes and Genomes (KEGG) enrichment results was high when NES was close to 2, and low when NES was close to −2. **(B)** Cluster analysis of combined protein and transcriptome data. Expression values were log2-transformed, and the mean value was subtracted; then the Hclust “Ward.D” method was used for cluster analysis. **(C)** KEGG enrichment analysis was performed for each protein class by Fisher’s exact test method, and the enrichment results for different classifications were combined. Then, the top 40 (*p*<0.05) significantly enriched functional classifications were identified. The horizontal axis shows fold enrichment after Log2 transformation, the vertical axis shows the functional classification, the bubble size represents the number of proteins, and the bubble color represents the value of *p* of enrichment significance.

We next performed Pearson GEST and KEGG pathway enrichment analysis of the DEGs and DEPs. The overall evaluation of Pearson correlation coefficient showed that the correlation coefficient between transcript and protein expression was 79.8%. The results showed that the vast majority of proteins were positively correlated with the transcript, but there were some cases of partial negative correlation. This study focused on proteins and transcripts with high Pearson correlation coefficients (Supplementary Figure S3). KEGG annotations were used as a gene set with known function, and the correlation coefficient between the input protein and the transcript was analyzed by GSEA to reveal the consistency and synergism of expression trends in metabolic pathways at the transcriptional and translational levels. For GEST, the gene transcription and protein expression trends such as carbon metabolism, fatty acid metabolism, and glyoxylate and dicarboxylate metabolism showed a clear positive correlation (Figure 6A; Supplementary Table S3). Cluster analysis combined genes in two dimensions of proteome and transcriptome expression. The study classifies the expression trends of proteins and transcripts at different developmental stages. From 4-mm spikelet to 8.5-mm spikelet with pollen development, proteins and transcripts in cluster 1 gradually increased. The proteins and transcripts in cluster 4 decreased gradually. Proteins and transcripts in cluster 2 were first enhanced and then decreased, and the expression level was the highest at 6-mm spikelets. The protein and transcript in cluster 3 decreased first and then increased, and the expression level was the lowest at 6-mm spikelet. The proteins of the four clusters were positively correlated with the transcripts. In clusters 5 and 6, transcripts showed a tendency for downregulation, whereas the corresponding proteins showed upregulation, this indicated strong post-translational regulation. The proteins of the two clusters were negatively correlated with the transcripts (Figure 6B). The study focuses on the genes of cluster 1 and cluster 2, cluster 1 reflects the important genes related to the transformation and synthesis of pollen energy substances (stage 12), while cluster 2 reflects the important genes related to anther elongation and tapetum formation (stage 6). We investigated the enriched KEGG pathways in these gene clusters and found that cluster 1 was enriched in carbon metabolism, glyoxylate and dicarboxylate metabolism, and fatty acid metabolism, consistent with the highest accumulation of sugars observed in the 8.5-mm spikelets. Cluster 2 was significantly enriched in fatty acid elongation, suggesting that the synthesis of long chain fatty acids was highest in 6-mm spikelets (Figure 6C; Supplementary Table S4).

### KEGG Pathways Enriched in Differentially Expressed Proteins

**Figure 7:**
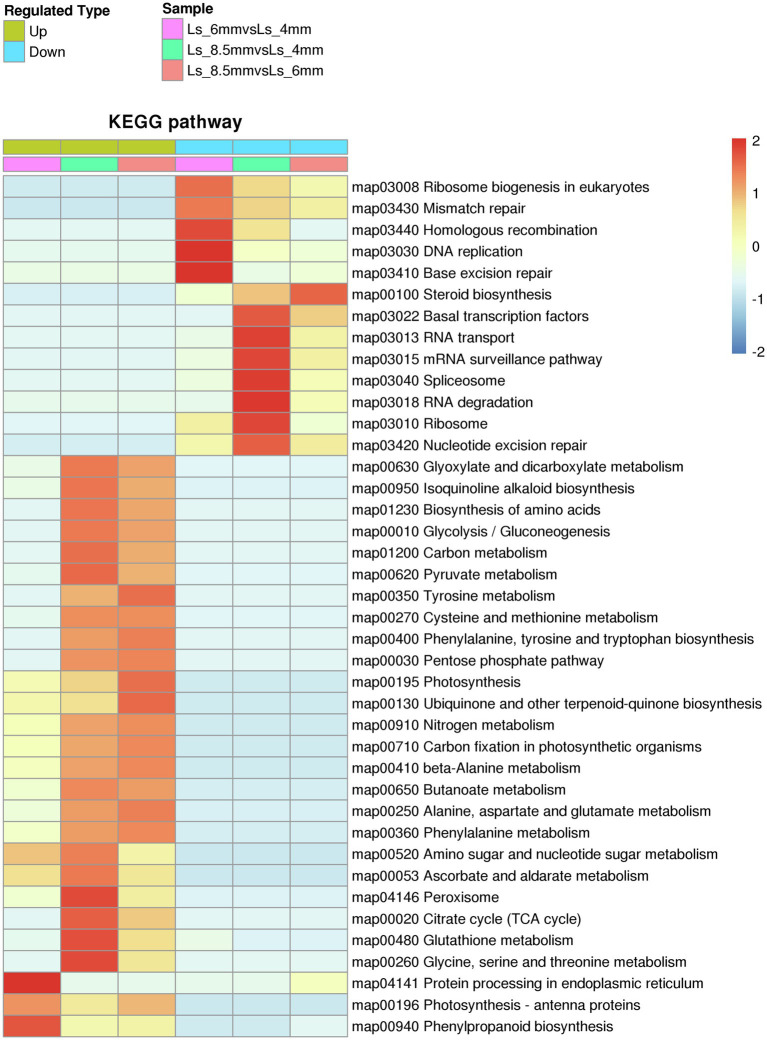
Kyoto Encyclopedia of Genes and Genomes pathways significantly enriched in sets of DEPs from three spikelet developmental stages. The enriched pathways included metabolic pathways by which fatty acids are converted to sugars.

Among the KEGG pathways significantly enriched in DEPs between different developmental stages were carbon metabolism and glyoxylate and dicarboxylate metabolism, both of which showed enrichment in Ls-6mm and Ls-8.5mm (Figure 7; Supplementary Table S5). In plant cells, fatty acids are broken down to acetyl-CoA by β-oxidation, a process that occurs in plastids and mitochondria (Hayashi et al., 1998; Zolman et al., 2001). In the glyoxylate cycle, glyoxylic acid is combined with acetyl-CoA to form malic acid, which is dehydrogenated to form oxaloacetic acid and then condensed with acetyl-CoA to form citric acid (Hu et al., 2012). Citric acid is isomerized to form isocitrate, and isocitrate lyase decomposes isocitrate into succinate and glyoxylic acid (Pan et al., 2019). Finally, succinic acid is transferred from the glyoxysome to the mitochondria (Kao et al., 2017). There, it enters the tricarboxylic acid cycle and is successively converted into fumaric acid, malic acid, and oxaloacetic acid (Hayashi and Nishimura, 2006), which produce glucose, fructose, and sucrose through gluconeogenesis (Nakamura et al., 1992). This is the process by which fatty acids are converted into sugars in plants.

**Figure 8:**
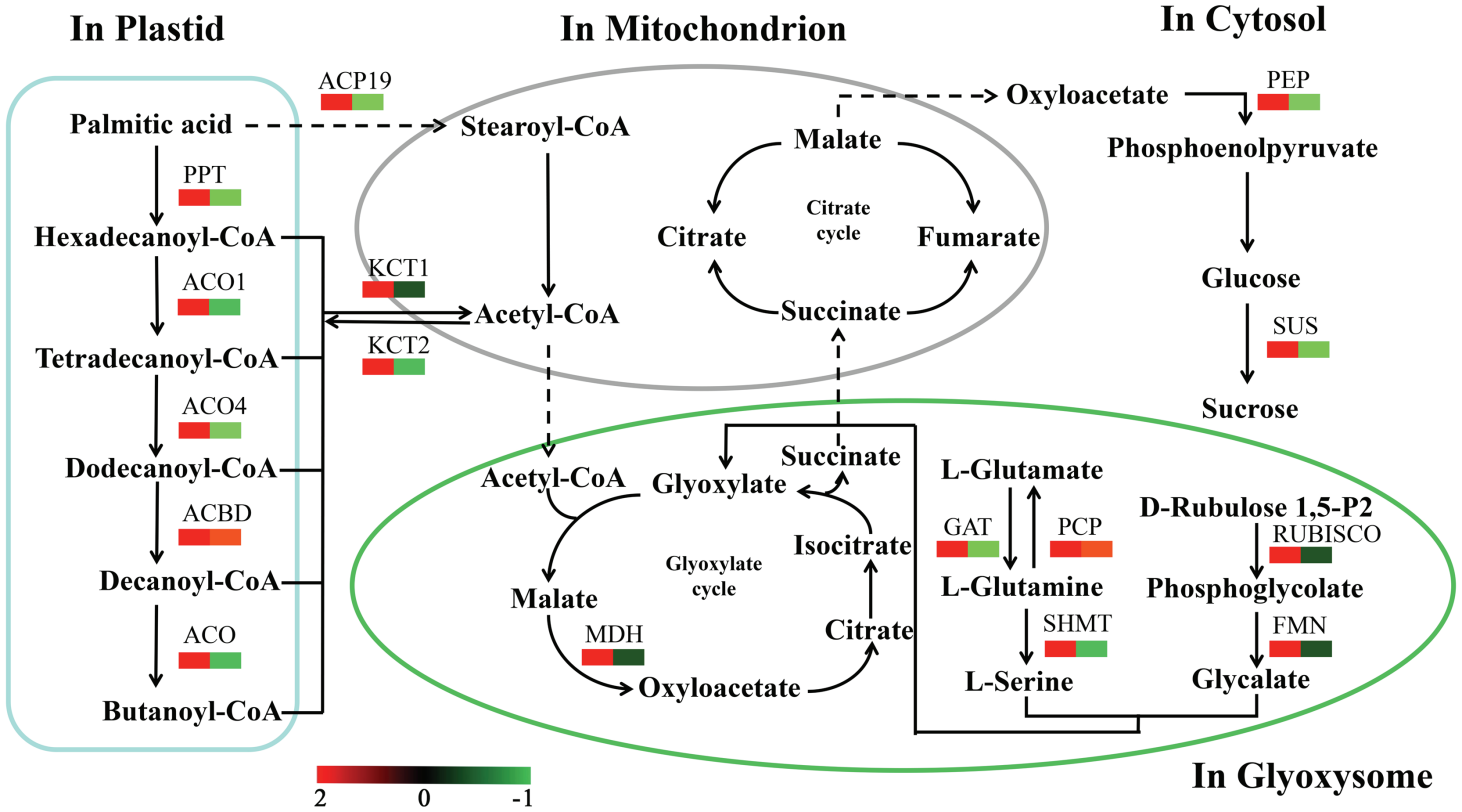
Changes in quantified proteins associated with the abundance of key enzymes during the different stages of pollen development in *O. longistaminata* (left box: Ls-8.5mm/Ls-6mm, right box: Ls-6mm/Ls-4mm).

We identified a number of enzymes associated with the conversion of fatty acids to sugars among the DEPs (Figure 8; Supplementary Table S6). Most of these fatty acid metabolism enzymes were upregulated from Ls-6mm to Ls-8.5mm. The informations of these key enzymes include enzyme symbol, EC number and fold change. They included palmitoyl protein thioesterase (PPT, EC:3.1.2.22, 2.77), acyl-coenzyme A oxidase 1 (ACO1, EC:1.3.3.6, 2.27), acyl-coenzyme A oxidase 4 (ACO4, EC:1.3.3.6, 2.31), acyl-CoA domain-containing protein (ACBD, EC:3.1.2.22, 1.52), acyl-coenzyme A oxidase (ACO, EC:1.3.3.6, 2.60), stearoyl-[acyl-carrier-protein] 9 (ACP9, EC:1.14.99.6, 2.91), 3-ketoacyl-CoA thiolase 1 (KCT1, EC:2.3.1.15, 1.62), and 3-ketoacyl-CoA thiolase 2 (KCT2, EC:2.3.1.16, 1.44). Also upregulated were enzymes of glyoxylate and dicarboxylate metabolism, such as glutamine synthetase (PCP, EC:6.3.1.2, 1.18), glutamine amidotransferase (GAT, EC:1.4.7.1, 2.26), malate dehydrogenase (MDH, EC:1.1.1.37, 6.98), serine hydroxymethyltransferase (SHMT, EC:2.1.2.1, 4.95), ribulose bisphosphate carboxylase (RUBISCO, EC:4.1.1.39, 14.70), and hydroxy acid dehydrogenase (FMN, EC:1.1.3.15, 24.04). We focused mainly on the gluconeogenesis enzymes of carbon metabolism: phosphoenolpyruvate carboxykinase (PEP, EC:6.3.1.2, 11.53) was significantly upregulated from Ls-6mm to Ls-8.5mm, as was sucrose synthase (SUS, EC:2.4.1.13, 1.19).

### Validation of Transcriptome and Proteome Data

**Figure 9:**
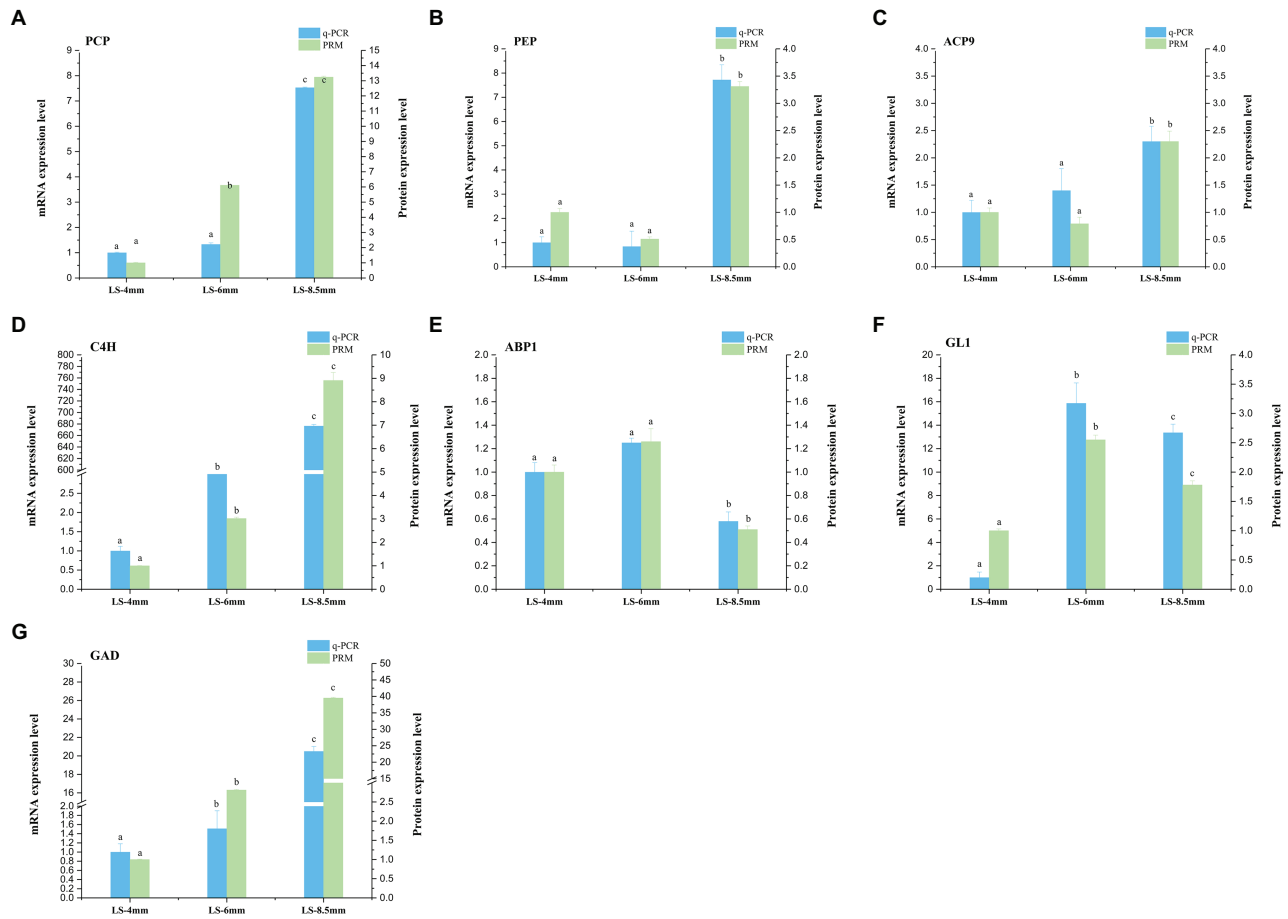
qRT-PCR and parallel reaction monitoring (PRM) of genes and proteins involved in in the metabolic pathways by which fatty acids are converted to sugars. Samples were obtained from 4-, 6-, and 8.5-mm spikelets in *O. longistaminata*. **(A–G)** represents qRT-PCR and PRM results of key enzymes PCP, PEP, ACP9, C4H, ABP1, GL1 and GAD, respectively. Relative gene expression and relative protein rates of each sample were compared to those in 4-mm spikelets. Actin was used as the internal reference gene with the 2^−ΔΔ*C*T^ method. Gene expression is shown in red and protein level in green. The data are means of three biological replicates, and different lowercase letters represent significant differences between stages within the same graph panel (*p*<0.05).

For transcriptome data validation, we selected genes encoding seven DEPs (PCP, PEP, SACP9, CAH9, ABP1, GL1, and GAD) involved in the metabolic pathways of fatty acid conversion to sugars. qRT-PCR was used to examine the corresponding mRNA expression levels of these selected proteins. qRT-PCR results for most of the selected proteins were consistent with the results of the transcriptomic data (Figure 9; Supplementary Table S7). To further validate the proteomic data, we performed PRM analysis on peptides representing seven proteins that were successfully quantified in the proteomic work (Supplementary Table S8). The PRM results also correlated well with the proteomics data (Figure 9). These results indicated that the transcriptome and proteome data were reasonable and reliable.

### *OlPCP* Gene Encodes a Endoplasmic Reticulum Localization Protein

**Figure 10:**
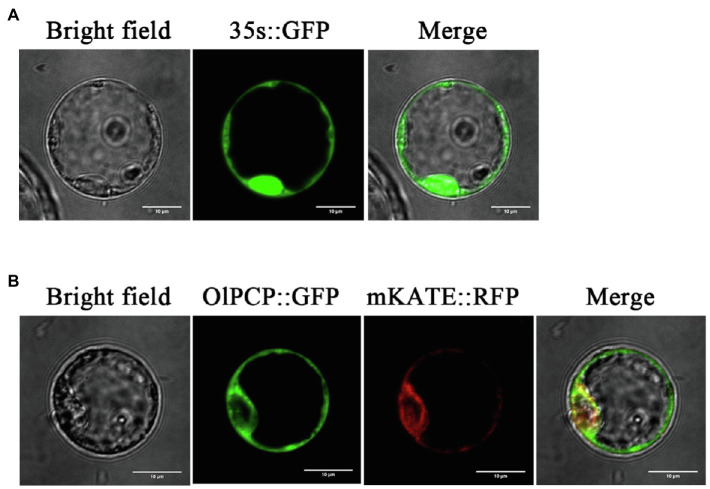
Subcellular localization of OlPCP protein. **(A)** Green fluorescent protein (GFP) protein signal promoted by 35S. **(B)** GFP represents OlPCP and red fluorescent protein (RFP) represents mKATE. Bar=10μm.

Glutamine synthetase (PCP, EC:6.3.1.2) was identified in the proteomics, and its gene ID (BGIOSGA009926) was also identified in the transcriptomics. The study named it *OlPCP* in *O. longistaminata*. KEGG annotated *OlPCP* gene, which was found to participates in glyoxylate and dicarboxylate metabolism during anther development. OlPCP is one of the key enzymes that convert fatty acids into sugars. For subcellular localization analysis, the protoplast transient gene expression vector pAN580 with the CaMV 35S promoter was used in this study. OlPCP was fused with GFP, while mKATE was fused with red fluorescent protein (RFP) as a endoplasmic reticulum localization signal. The GFP signal of the OlPCP protein colocalized with the RFP signal of the mKATE protein in the endoplasmic reticulum, while the signal of 35s::GFP was detected throughout the cell. This observation revealed the endoplasmic reticulum localization of OlPCP (Figure 10). OlPCP synthesized in the endoplasmic reticulum and eventually participated in glyoxylate cycle.

## Discussion

### Transcriptomic and Proteome Characteristics of Pollen Development in *Oryza longistaminata*

Our transcriptomic and proteomic study of different pollen development of *O. longistaminata* showed that there was substantial concordance between the proteins and transcripts. In fact, transcripts were detected for 27.4% of the proteins. In this study, the proteome data were much smaller than the transcriptome data, which may be due to the existence of many unanalyzed proteins in *O. longistaminata* (Zhang et al., 2015). Some proteins that were detected by proteomics analysis were not detected in the mRNA analysis in rice, which was attributed to mRNA half-life and stability (Chen et al., 2019). However, there is a high concordance (79.8%) between the identified proteins and the transcriptome. A modest concordance between mRNA levels and proteins in Human and *Arabidopsis* (Fathi et al., 2009; Lan et al., 2011). Partial discordance between mRNA and proteins was determined by translational and post-translational processes. Since the difference between DEGs and DEPs may come from mRNA synthesis rate, post-transcriptional regulation and other translation and post-translation mechanisms (Nakaminami et al., 2014), and top20 differential proteins were screened to understand the function of an enzyme. Many of these proteins are involved in various metabolic pathways, including the biosynthesis of secondary metabolites, fatty acid and glyoxylic acid metabolism (Chen et al., 2019). The glyoxylate acid cycle converts fatty acids to sugars during seed germination of oil plants such as peanut, rapeseed, and cottonseed (Smith, 2002). It was reported that as the activities of glyoxylate acid metabolism-related enzymes increased in rapeseed, the pollen lipid content decreased and the sugar content increased significantly (Zhang et al., 1994). Our study is the first to show a similar pathway in *O. longistaminata*, and enzymes related to the conversion of fatty acids to sugars (which are mainly involved in fatty acid metabolism, the glyoxylic acid cycle, and gluconeogenesis) affect pollen viability. These new data provide theoretical guidance for making use of viable *O. longistaminata* pollen in rice breeding.

### Confirmation of Pollen Viability and the Main Factors That Influence Pollen Germination

Due to self-incompatibility, we had to measure pollen activity separately (Franklin-Tong et al., 2002). The results of I_2_-KI staining, TTC staining, pollen tube growth observation, and pollen tube imprinting showed that *O. longistaminata* had good pollen viability. The pollen development of *O. longistaminata* is different from that of indica/japonica rice, and we therefore performed cytological analysis of anthers from spikelets of different lengths (Tsou et al., 2015). We found that the microspore undergoes the first mitotic division with asymmetric cell division, the microspores are falcate in shape, and starch accumulates inside the microspore. Then the microspore undergoes the second mitosis and divides into two sperm cells. At this point, starches and lipids have accumulated completely, and the spikelet is 8.5mm in length and at stage 11–12 (Li et al., 2010, 2011a,b; Li and Zhang, 2010). Sucrose provides energy for pollen germination, maintains the osmotic balance between pollen and the culture medium, and prevents damage and rupture of the pollen and pollen tube. In a previous report, a sucrose concentration of 10–20% produced the most plant pollen germination *in vitro* (Yin and Zhao, 2005). In this study, optimum germination required more energy (40% sucrose concentration), and SUS was significantly upregulated. These results suggest that more sucrose is synthesized in plants to provide energy for pollen germination in *O. longistaminata*.

### Enzymes Related to the Glyoxylate Cycle During Pollen Development

The glyoxysome is a type of peroxisome (Breidenberg and Beevers, 1967). The glyoxylate cycle enzymes CS, ICL, MS and MDH are localized in the glyoxysome, together with catalase and enzymes of fatty acid β-oxidation (Nishimura et al., 1986). In this study, MDH was successfully identified, quantified, and found to increase in abundance from the 4-mm spikelet to the 8.5-mm spikelet. These data suggest that MDH plays an important role in the conversion of fatty acids to sugars. The direction of action of MDH was OAA reduction rather than malate oxidation, indicating the important role of cytoplasmic MDH in the glyoxylate cycle (Minard and McAlister-Henn, 1991). On the one hand, glutamate is converted to glutamine by the enzyme GAT, glutamine is converted to serine by SHMT, and the enzymes GAT and SHMT were upregulated in 8.5-mm spikelets only stage. On the other hand, ribulose 1,5-bisphosphate is converted to phosphoglycolate by the enzyme RUBISCO, phosphoglycolate is converted to glycolate by FMN, and the enzymes RUBISCO and FMN were upregulated from 6-mm spikelets to 8.5-mm spikelets. The reaction products of these two metabolic pathways, serine and glycolate, are synthesized into glyoxylate, which is used as the raw material for the glyoxylate cycle (Graham et al., 1994; Chen et al., 2000). In oil crops, the expression of glyoxylate cycle-related genes is related to the developmental stage in which pollen lipid content declines, and fatty acid degradation is related to these genes during seed germination. We speculate that these genes may have similar functions in pollen development and seed development (Zhang et al., 1993, 1994, 1996).

### Enzymes Related to Fatty Acid Degradation During Pollen Development

Fatty acids degrade from palmitic acid to form hexadecanoyl-CoA, tetradecanoyl-CoA, dodecanoyl-CoA, decanoyl-CoA, and butanoyl-CoA, and almost all of the fatty acid degradation-related enzymes that catalyze carbon chain reductions were upregulated in 6-mm spikelets, indicating rapid degradation of fatty acids at about the 6-mm spikelet stage. The enzymes PPT, ACBD, and ACO play essential roles in fatty acid degradation during pollen development of *O. longistaminata*. Fatty acids must form acyl-CoA with CoA before entering the mitochondrion. Acyl-CoA enters the mitochondrion and forms acetyl-CoA by fatty acid β-oxidation, and the KCT1 enzyme related to fatty acid oxidation was significantly up-regulated (Laus et al., 2011). In addition, palmitic acid is converted to stearoyl-CoA by the enzyme ACP19, and stearoyl-CoA is degraded to acetyl-CoA through a metabolic pathway similar to that of saturated fatty acids. The rate of fatty acid β-oxidation is controlled by the entry rate of fatty acid CoA into the mitochondria. The glyoxylate cycle consumes acetyl-CoA, which accelerates fatty acid β-oxidation and then promotes the entry of fatty acid CoA into the mitochondria (Diakou et al., 2002; Fan et al., 2017).

### Enzymes Related to Gluconeogenesis and Sucrose Synthesis During Pollen Development

The distribution patterns of some hydrolases in germinating pine pollen indicate that enzymes related to gluconeogenesis significantly increase during different pollen germination periods (0–75h; Malik et al., 1976; Singh et al., 1976). Fats and amino acids are converted very efficiently into sugars by gluconeogenesis in pine pollen, and this ancient metabolic pathway was found in gymnosperms and later in angiosperms such as oil crops, soybeans, and corn (Malik et al., 1978). Here, PEP was successfully identified, quantified, and found to be upregulated in 8.5-mm spikelets only. Phosphoenolpyruvate was formed in the cytoplasm from oxaloacetate by the PEP reaction after the degradation of surface fatty acids through the glyoxylic acid cycle and the TCA cycle. Glucose was synthesized into sucrose (Nakamura et al., 1992), and SUS was also upregulated in 8.5-mm spikelets only. In particular, these results show that regulation of the sucrose synthesis reaction was consistent with that of the gluconeogenesis reaction. Ultimately, this study found a new pathway in which fatty acids can be converted into sugars during pollen development in *O. longistaminata*, and this process may be closely related to pollen viability.

## Data Availability Statement

The original contributions presented in the study are publicly available. This data can be found here: National Center for Biotechnology Information (NCBI) BioProject database under accession number GSE181045.

## Author Contributions

HH and XP conceived and designed this research as well as wrote the manuscript. YS conducted the experiments and wrote the manuscript. YS, XW, ZC, BL, and LO performed the experiments. All authors contributed to the article and approved the submitted version.

## Funding

This research was supported by the National Key Research and Development Program of China (2016YFD0101104) and Major Project of Jiangxi Provincial Department of Science and Technology (S2016NYZPF0256).

## Conflict of Interest

The authors declare that the research was conducted in the absence of any commercial or financial relationships that could be construed as a potential conflict of interest.

## Publisher’s Note

All claims expressed in this article are solely those of the authors and do not necessarily represent those of their affiliated organizations, or those of the publisher, the editors and the reviewers. Any product that may be evaluated in this article, or claim that may be made by its manufacturer, is not guaranteed or endorsed by the publisher.

## Footnotes

^1^ ftp://ftp.ensembl.org/pub/release-41/fasta/oryzalongistaminata/dna/
