# Supplementary material for: Quantitative Proteomics and Transcriptomics Reveals Differences in Proteins During Anthers Development in Oryza longistaminata
Source: Front Plant Sci. 2021 Nov 19;12:744792. doi: 10.3389/fpls.2021.744792 (PMC8640343; doi:10.3389/fpls.2021.744792)
Supplement: Supplementary file 1 [file Data_Sheet_1.doc]

| 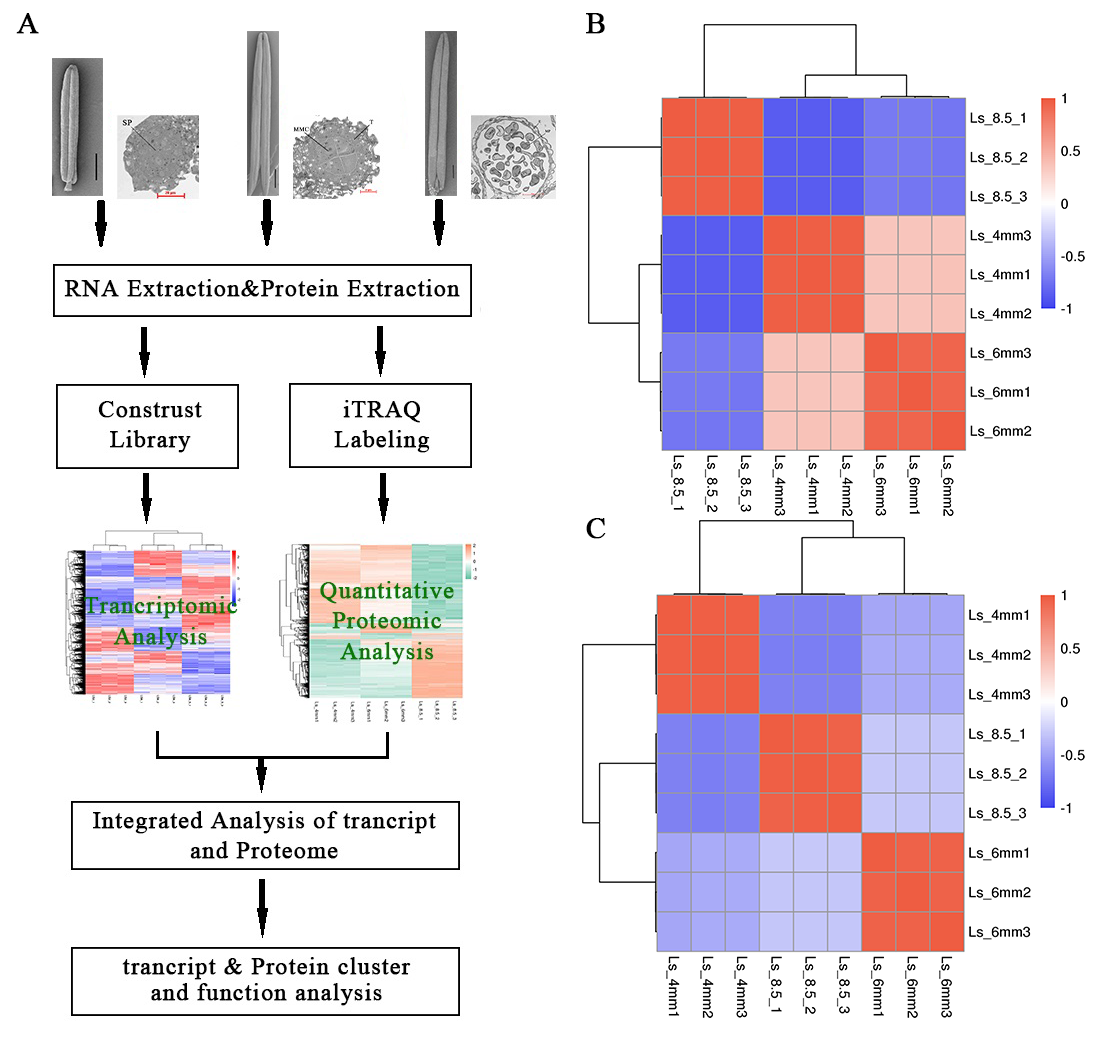 |
| --- |
| **Figure S1.** **Clustering heat map of pearson correlation coefficient.** (A) Schematic workflow of transcriptome and proteome analysis over the course of pollen development in *O. longistaminata*. (B) Clustering heat map of proteomic correlation coefficients; (C) Clustering heat map of transcriptome correlation coefficients. After log2 conversion of the quantitative value and subtracting the mean value, the COR function in R was used to calculate the Pearson correlation coefficient between two samples. After that, the heatmap of PheatMap package in R was used to draw a visual display of the correlation coefficient. The darker the red, the more positive the correlation, and the darker the blue, the more negative the correlation. The better the reproducibility between samples, the redder the color. |

| **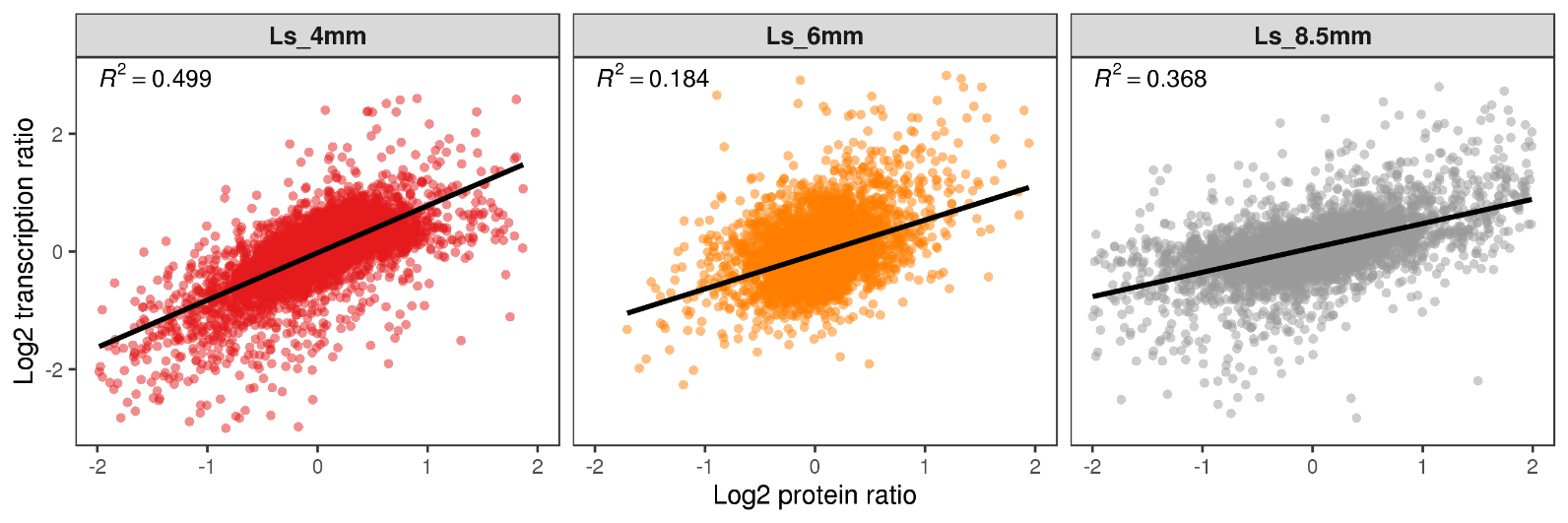** |
| --- |
| **Figure S2.** **Scatter plot of transcripts and their corresponding proteins.** According to the above corresponding relationship between protein and transcriptome ID, the protein expression level and mRNA expression level were combined to draw the scatter plot of protein and mRNA expression level. In order to unify the definition of expression levels, log2 conversion of both protein and transcriptome expression levels is required before drawing the scatter plot, and the mean value is subtracted. In the figure, protein expression is shown on the horizontal axis and transcript expression is shown on the vertical axis. For different samples, draw a scatter chart and add a trend line. |

| **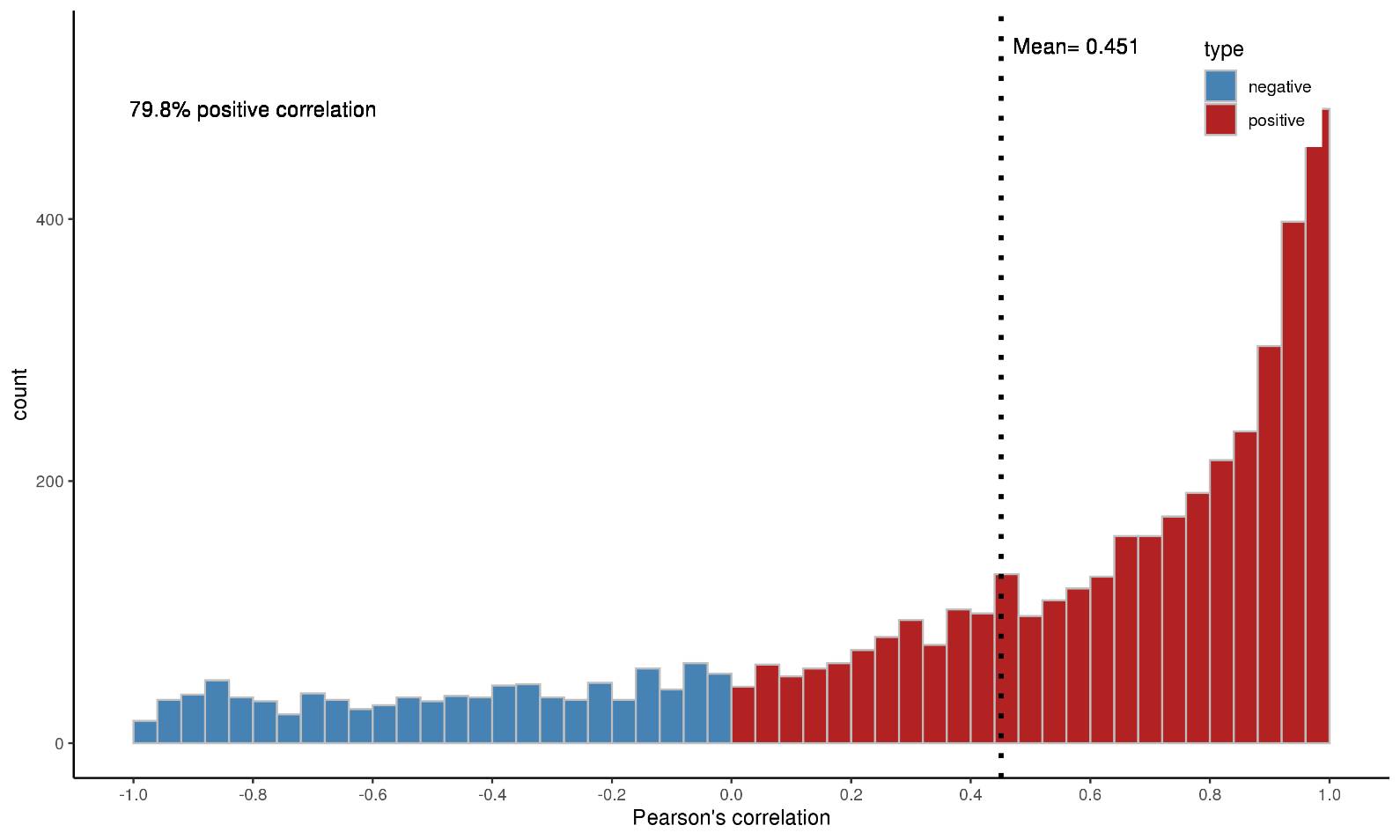** |
| --- |
| **Figure S3.** **Cumulative distribution of Pearson correlation coefficient between transcriptome and proteome quantification.** The Pearson correlation coefficients of each protein and the corresponding transcript were calculated, and the Pearson correlation coefficients were calculated and the histogram was drawn. The abscissor represents the Pearson correlation coefficient, and the ordinate represents the number of proteins or transcripts under the Pearson correlation coefficient. Pearson correlation coefficient greater than 0 (positive correlation between protein and transcript) is shown in red, and Pearson correlation coefficient less than 0 (negative correlation between protein and transcript) is shown in blue. |

Table S1. Rusult information of fatty acid content at three developmental stages in *O. longistaminata*.

| Fatty acid name | Ls-4mm | Ls-6mm | Ls-8.5mm |
| --- | --- | --- | --- |
| Linoleic acid(18:2^-Δ9C ,12C^) | 33.65±0.22 | 30.62±0.31 | 18.59±0.03 |
| Oleic acid(18:1^-Δ9C^) | 9.48±0.1 | 12.01±0.07 | 7.2±0.02 |
| Palmitic acid(16:0) | 25.72±0.24 | 24.09±0.29 | 18.67±0.05 |
| Decanoic acid(10:0) | 3.47±0.09 | 2.72±0.04 | 2.32±0.05 |
| Octanoic acid(8:0) | 0.83±0.21 | 0.83±0.15 | 0.37±0.26 |
| Butanoic acid(4:0) | 7.72±0.07 | 6.05±0.07 | 4.07±0.07 |

Table S2. Rusult information of sugar components at three developmental stages in *O. longistaminata*.

| Sugar name | Ls-4mm | Ls-6mm | Ls-8.5mm |
| --- | --- | --- | --- |
| sucrose | 23.56±1.02 | 37.57±0.56 | 60.33±1.32 |
| glucose | 8.06±0.35 | 10.21±0.67 | 19.82±0.88 |
| fructose | 11.36±0.42 | 15.97±0.73 | 26.89±1.02 |

Table S3. GSEA result information of KEGG pathway based on quantitative correlation coefficient of transcriptome and proteome in *O. longistaminata*.

| KEGG pathway name | SIZE | NES | NOM p-val |
| --- | --- | --- | --- |
| Nitrogen metabolism | 13 | 0.70 | 0 |
| Tryptophan metabolism | 21 | 0.58 | 0 |
| Lysine degradation | 17 | 0.60 | 0 |
| Phenylalanine metabolism | 16 | 1.99 | 0 |
| N-glycan biosynthesis | 20 | 1.97 | 0.001106195 |
| Glyoxylate and dicarbocxylate metabolism | 58 | 1.95 | 0 |
| Various types of n-glycan biosynthesis | 22 | 1.87 | 0 |
| Phenylpropanoid biosynthesis | 63 | 1.85 | 0 |
| Butanoate metabolism | 21 | 1.84 | 0.004524887 |
| Arginine and proline metabolism | 24 | 1.69 | 0.005464481 |
| Stilbenoid, diarylheptanoid and gingerol biosynthesis | 8 | 1.66 | 0.021518987 |
| Pryuvate metabolism | 71 | 1.64 | 0.003033367 |
| Beta-alanine metabolism | 25 | 1.61 | 0.014223195 |
| Carbon metabolism | 204 | 1.61 | 0 |
| Alanine, aspartate and glutamate metabolism | 39 | 1.60 | 0.006335797 |
| Glycolysis/Gluconeogenesis | 101 | 1.60 | 0.002020202 |
| Valine, leucine and isoleucine degradation | 33 | 1.60 | 0.012834225 |
| Selenocompound metabolism | 15 | 1.60 | 0.016627079 |
| Citrate cycle(TCA cycle) | 51 | 1.60 | 0.007209063 |
| Propanoate metabolism | 26 | 1.58 | 0.02826087 |
| Flavonoid biosynthesis | 21 | 1.56 | 0.030371204 |
| Glycine, serine and threonine metabolism | 47 | 1.56 | 0.014705882 |
| Metabolic pathways | 1072 | 1.56 | 0 |
| Biosynthesis of secondary metabolites | 569 | 1.55 | 0 |
| Carbon fixation in photosynthetic organisms | 57 | 1.54 | 0.012219959 |
| Cyanoamino acid metabolism | 23 | 1.54 | 0.03080308 |
| Phenylalanine, tyrosine and tryptophan biosynthesis | 31 | 1.54 | 0.031453364 |
| Steroid biosynthesis | 11 | 1.52 | 0.034739453 |
| Taurine and hypotaurine metabolism | 5 | 1.52 | 0.042876903 |
| Cysteine and methionine metabolism | 61 | 1.51 | 0.018386109 |
| Isoquinoline alkaloid biosynthesis | 8 | 1.51 | 0.04713376 |
| Peroxisome | 54 | 1.49 | 0.024640657 |
| Glutathione metabolism | 59 | 1.48 | 0.029501526 |
| Fructose and mannose metabolism | 43 | 1.47 | 0.043933053 |
| Synthesis and degradation of ketone bodies | 5 | 1.46 | 0.049792532 |
| Fatty acid metabolism | 50 | 1.45 | 0.043568466 |
| Diterpenoid biosynthesis | 2 | 1.44 | 0.036977492 |
| Biosynthesis of amina acids | 174 | 1.39 | 0.009 |
| Amino sugar and nucleotide sugar metabolism | 70 | 1.38 | 0.03560529 |
| Ubiquitin mediated proteolysis | 37 | -2.39 | 0 |
| Proteasome | 44 | -1.72 | 0.025 |
| Ribosome biogenesis in eukaryotes | 31 | -1.63 | 0.012820513 |
| RNA transport | 101 | -1.31 | 0 |

Table S4. Result information of KEGG pathway enrichment(cluster1) in *O. longistaminata*.

| Terms | KEGG pathway name | Mapping | Fold enrichment | p value |
| --- | --- | --- | --- | --- |
| map00196 | Photosynthesis | 10 | 9.86 | 8.53E-09 |
| map00195 | Photosynthesis | 19 | 5.03 | 2.47E-09 |
| map00020 | Citrate cycle (TCA cycle) | 23 | 4.8 | 1.43E-10 |
| map00630 | Glyoxylate and dicarboxylate metabolism | 35 | 4.74 | 2.17E-13 |
| map00710 | Carbon fixation in photosynthetic organisms | 29 | 4.51 | 2.87E-12 |
| map00030 | Pentose phosphate pathway | 17 | 3.87 | 1.11E-06 |
| map00400 | Phenylalanine, tyrosine and tryptophan biosynthesis | 15 | 3.7 | 8.60E-06 |
| map01230 | Biosynthesis of amino acids | 63 | 2.84 | 4.7913E-14 |
| map00220 | Arginine biosynthesis | 12 | 3.43 | 0.000145307 |
| map00620 | Pyruvate metabolism | 28 | 3.38 | 9.04E-09 |
| map00260 | Glycine, serine and threonine metabolism | 23 | 3.34 | 2.39E-07 |
| map01200 | Carbon metabolism | 92 | 3.82 | 1.8082E-30 |
| map00051 | Fructose and mannose metabolism | 15 | 3.13 | 6.81E-05 |
| map00280 | Valine, leucine and isoleucine degradation | 15 | 2.96 | 0.0001337 |
| map00010 | Glycolysis / Gluconeogenesis | 32 | 2.82 | 7.16E-08 |
| map00250 | Alanine, aspartate and glutamate metabolism | 17 | 2.87 | 7.06E-05 |
| map00270 | Cysteine and methionine metabolism | 28 | 2.81 | 5.50E-07 |
| map00190 | Oxidative phosphorylation | 23 | 2.5 | 3.88E-05 |
| map01100 | Metabolic pathways | 358 | 1.68 | 1.51E-48 |
| map01110 | Biosynthesis of secondary metabolites | 186 | 1.61 | 1.0919E-07 |

Table S5. Peptide sequences with post translational modifications identified involved in sucrose accumulation and fatty acid degradation in *O. longistaminata*.

| Gene ID | Strategy | Annotation | Peptide sequences with post translational modifications identified by MS |
| --- | --- | --- | --- |
| **Glyoxylate and dicarboxylate metabolism** | | | |
| BGIOSGA001752 | RNA-seq/MS | Dihydrolipoyl dehydrogenase | EACLQTFTK |
| BGIOSGA035778 | RNA-seq/MS | Malate dehydrogenase | ANTFVAEVLGLDPR |
| BGIOSGA040203 | RNA-seq/MS | Ribulose bisphosphate carboxylase large chain | DDFIEKDR |
| BGIOSGA007627 | RNA-seq/MS | Glycine cleavage system H protein | VEGDSATVGITDHAQHHLGDVVYVELPEVGSSVSQGK |
| BGIOSGA023636 | RNA-seq/MS | Catalase | WVDALSDPR |
| BGIOSGA002081 | RNA-seq/MS | Hydroxyphenylpyruvate reductase | EVMEALGPR |
| BGIOSGA004108 | RNA-seq/MS | Malate dehydrogenase | GLNGVPDVVECSFVQSTVTELPFFASK |
| BGIOSGA011520 | RNA-seq/MS | Catalase | GPILLEDYHLVEK |
| BGIOSGA011492 | RNA-seq/MS | Aconitate hydratase | GPMLQGVK |
| BGIOSGA007840 | RNA-seq/MS | Citrate synthase | HLASSGVDVFTALSGAVGALYGPLHGGANEAVLK |
| BGIOSGA005667 | RNA-seq/MS | Glutamine synthetase | ASLTDLVNLNLSDTTEK |
| BGIOSGA013686 | RNA-seq/MS | FMN hydroxy acid dehydrogenase domain-containing protein | FVLPPYLTLK |
| BGIOSGA035211 | RNA-seq/MS | Serine hydroxymethyltransferase | GLDLPSGGHLTHGYYTAGGK |
| BGIOSGA013524 | RNA-seq/MS | Serine hydroxymethyltransferase | GLELIPSENFTSVSVMQAVGSVMTNK |
| BGIOSGA007314 | RNA-seq/MS | Hydroxypyruvate reductase | AATMEDVLR |
| BGIOSGA019228 | RNA-seq/MS | Dihydrolipoyl dehydrogenase | AEEDGVACVEFIAGK |
| BGIOSGA038154 | RNA-seq/MS | Ribulose bisphosphate carboxylase small chain | AYPDAFVR |
| BGIOSGA002627 | RNA-seq/MS | Acetyl-CoA acetyltransferase, cytosolic | DSGAFSWEIAPVEISSGR |
| BGIOSGA009738 | RNA-seq/MS | Malate dehydrogenase | DVNVPVVGGHAGVTILPLLSQVHPPCSFTPDEISYLTK |
| BGIOSGA006961 | RNA-seq/MS | Citrate synthase | GMTGMLWETSLLDPDEGIR |
| BGIOSGA017166 | RNA-seq/MS | Aminomethyltransferase (Fragment) | KAEGGFLGADVILK |
| BGIOSGA025165 | RNA-seq/MS | FMN hydroxy acid dehydrogenase domain-containing protein | AASAAGTIMTLSSWATSSVEEVASTGPGIR |
| BGIOSGA035391 | RNA-seq/MS | Citrate synthase | AIDALPVTAHPMTQFTTGVMALQVESEFQK |
| BGIOSGA020897 | RNA-seq/MS | Glycine cleavage system P protein | FDAGFTESEMIEHMQR |
| BGIOSGA004313 | RNA-seq/MS | Glycine cleavage system P protein | FDAGFTESQMIDHMQR |
| BGIOSGA001365 | RNA-seq/MS | Glyoxylate/succinic semialdehyde reductase 2, chloroplastic | GYVDVSTVDAATSK |
| BGIOSGA011196 | RNA-seq/MS | Glutamine synthetase | HETADINTFK |
| BGIOSGA014826 | RNA-seq/MS | OSIGBa0130B08.3 protein | IELKPGFYMEHDK |
| BGIOSGA033278 | RNA-seq/MS | Glycine cleavage system H protein | LSETPGLINSSPYEDGWMIK |
| BGIOSGA024963 | RNA-seq/MS | Aminotran_1_2 domain-containing protein | NVVCNFTEGAMYSFPQIR |
| BGIOSGA026932 | RNA-seq/MS | Malate dehydrogenase | RIQNAGTEVVEAK |
| BGIOSGA004733 | RNA-seq/MS | Malate dehydrogenase | TLVEAVADNCPEAFIHIISNPVNSTVPIAAEVLK |
| BGIOSGA016334 | RNA-seq/MS | Acetyl-coenzyme A synthetase | CAEAAVVGIDHEVK |
| BGIOSGA010892 | RNA-seq/MS | AMP-binding enzyme family protein, expressed | DEWQSIALGYTSGTTSSPK |
| BGIOSGA023784 | RNA-seq/MS | Glutamine amidotransferase type-2 domain-containing protein | EATLQSPVWR |
| **Fatty acid metabolism** | | | |
| BGIOSGA002627 | RNA-seq/MS | Acetyl-CoA acetyltransferase, cytosolic 2 OS=Zea mays | EEQDLYAIQSNER |
| BGIOSGA028458 | RNA-seq/MS | Enoyl-[acyl-carrier-protein] reductase | KLPDGSLMEIVK |
| BGIOSGA012483 | RNA-seq/MS | Enoyl-CoA hydratase/isomerase family protein, expressed | PMMVSLAAAFR |
| BGIOSGA021732 | RNA-seq/MS | PKS_KS domain-containing protein | QHEVNVGISNSFGFGGHNSVVVFAPFKP |
| BGIOSGA015217 | RNA-seq/MS | Stearoyl-[acyl-carrier-protein] 9-desaturase 5, chloroplastic | SWQPQDFLPDPSSDGFYDEVK |
| BGIOSGA027438 | RNA-seq/MS | FabA domain-containing protein | AAGDGQAVEAQEALPIEK |
| BGIOSGA030415 | RNA-seq/MS | Enoyl-[acyl-carrier-protein] reductase | KLPDGSLMEITK |
| BGIOSGA015012 | RNA-seq/MS | 3-oxoacyl-[acyl-carrier-protein] synthase | NDDPETASRPWDK |
| BGIOSGA033076 | RNA-seq/MS | 3-ketoacyl-CoA thiolase 2, peroxisomal, putative, expressed | PGTSLAVLSK |
| BGIOSGA012826 | RNA-seq/MS | PKS_KS domain-containing protein | RGETDVMLCGGSDAPIIPIGLGGFVACR |
| BGIOSGA019926 | RNA-seq/MS | FabA domain-containing protein | VIDYKPGEYAVGIK |
| BGIOSGA031709 | RNA-seq/MS | Very-long-chain aldehyde decarbonylase | VVSLGLLNQAHTLNK |
| BGIOSGA021280 | RNA-seq/MS | Acyl-coenzyme A oxidase | WLSDTENYVIK |
| BGIOSGA021279 | RNA-seq/MS | Acyl-coenzyme A oxidase | AMEESPLFCQR |
| BGIOSGA005359 | RNA-seq/MS | 3-ketoacyl-CoA thiolase-like protein | AQDCLLPMGITSENVAHR |
| BGIOSGA004497 | RNA-seq/MS | Long-chain-fatty-acid-CoA ligase-like protein | EGTVGKPLPR |
| BGIOSGA011650 | RNA-seq/MS | Palmitoyl protein thioesterase containing protein, expressed | ETAWFGYYPDGGFDPVLPPQK |
| BGIOSGA001018 | RNA-seq/MS | AMP-binding domain-containing protein | EVLVDGWFHTGDIGEWQSDGSMK |
| BGIOSGA019609 | RNA-seq/MS | AMP-binding domain-containing protein | FVLCGGAPLSSDTQR |
| BGIOSGA036643 | RNA-seq/MS | AMP-binding domain-containing protein | FYPSDVYISYLPLAHIYER |
| **Glycolysis / Gluconeogenesis** | | | |
| BGIOSGA011083 | RNA-seq/MS | Phosphoenolpyruvate carboxykinase | GILSLHSGCNMGK |
| BGIOSGA010570 | RNA-seq/MS | Sucrose synthase | GMLQAHQIIAEYNNAISEADR |
| BGIOSGA008865 | RNA-seq/MS | Phosphoribulokinase | HADFPGSNNGTGLFQTIVGLK |
| BGIOSGA000792 | RNA-seq/MS | Phosphoenolpyruvate carboxylase | HTDVMDAITQYLGIGSYR |
| BGIOSGA026849 | RNA-seq/MS | Glutamate decarboxylase | ILHELDAHSAQVLK |
| BGIOSGA029976 | RNA-seq/MS | Glucose-6-phosphate 1-epimerase | IVISNANWSDAVLWNPHLQMEACYK |
| BGIOSGA029780 | RNA-seq/MS | Dihydrolipoamide acetyltransferase | IVSWTAAEGDR |
| BGIOSGA022643 | RNA-seq/MS | Glucose-6-phosphate isomerase | MEGKPVQGFNSSTASLLTR |
| BGIOSGA020215 | RNA-seq/MS | Phosphotransferase | SDKLPLSEFDK |
| BGIOSGA001898 | RNA-seq/MS | Pyruvate kinase | SITASAPIPMSPLESLASSAVR |
| BGIOSGA007341 | RNA-seq/MS | Acetyltransferase component of dehydrogenase complex | VIPGSVDGQYEFGSFMSATMSCDHR |
| BGIOSGA001752 | RNA-seq/MS | Dihydrolipoyl dehydrogenase | VPYTAGIGLESVGVETDK |
| BGIOSGA002932 | RNA-seq/MS | ATP-dependent 6-phosphofructokinase | GGLLEFIEK |
| BGIOSGA011109 | RNA-seq/MS | Enolase 2, putative, expressed | GNPTVEVDICCSDGTFAR |
| BGIOSGA031063 | RNA-seq/MS | Pyruvate dehydrogenase E1 component subunit beta | GPNGAAAGVGAQHSQCYAAWYAHVPGLK |
| BGIOSGA029371 | RNA-seq/MS | Triosephosphate isomerase | HVIGEDDQFIGK |
| BGIOSGA000849 | RNA-seq/MS | Phosphotransferase | LAAAGIYGILK |
| BGIOSGA005654 | RNA-seq/MS | Pyruvate dehydrogenase E1 component subunit alpha | LILAHDLATAAELK |
| BGIOSGA005972 | RNA-seq/MS | PKS_ER domain-containing protein | SDVTQWQSTAQTDLFPR |
| BGIOSGA034421 | RNA-seq/MS | Fructose-bisphosphate aldolase | TFEVAQK |
| BGIOSGA032274 | RNA-seq/MS | Aldose 1-epimerase | TLDLWTDAPGMQFYTANYVDGITGK |
| BGIOSGA029064 | RNA-seq/MS | Pyruvate dehydrogenase E1 component subunit beta | VLDTPITEAGFTGIGVGAAYQGLR |
| BGIOSGA022101 | RNA-seq/MS | Acetyltransferase component of dehydrogenase complex | ADIEDYLASVAK |
| BGIOSGA017793 | RNA-seq/MS | Phosphoglycerate kinase | ADLNVPLDDNQNITDDTR |
| BGIOSGA021193 | RNA-seq/MS | Dihydrolipoamide acetyltransferase | DVQVGQPIAVTVEDLEDIK |
| BGIOSGA029762 | RNA-seq/MS | PFK domain-containing protein | FSLPHITDIYDLKPR |
| BGIOSGA011043 | RNA-seq/MS | FBPase domain-containing protein | QEQAGTIDGEMTIVLASISTACK |
| BGIOSGA013652 | RNA-seq/MS | Glucose-6-phosphate isomerase | PVLGFNSSTTSLLTR |
| BGIOSGA004775 | RNA-seq/MS | Triosephosphate isomerase | IIYGGSVNGANCK |
| BGIOSGA023247 | RNA-seq/MS | Fructose-bisphosphate aldolase | ENVAAAQAAFLAR |
| BGIOSGA019844 | RNA-seq/MS | Fructose-bisphosphate aldolase | EGGVLPGIK |
| BGIOSGA022810 | RNA-seq/MS | Pyrophosphate--fructose 6-phosphate 1-phosphotransferase | DKNHGVVLIPEGLVESIPELYALLQEIHGLHDK |
| BGIOSGA016334 | RNA-seq/MS | Acetyl-coenzyme A synthetase | CAEAAVVGIDHEVK |
| BGIOSGA010892 | RNA-seq/MS | AMP-binding enzyme family protein, expressed | ANAEAFAGGWYHSGDLGVK |
| BGIOSGA004691 | RNA-seq/MS | 2,3-bisphosphoglycerate phosphoglycerate mutase | AHGTAVGLPSEDDMGNSEVGHNALGAGR |
| BGIOSGA008564 | RNA-seq/MS | Glyceraldehyde-3-phosphate dehydrogenase | AGIALSDTFVK |
| BGIOSGA019228 | RNA-seq/MS | Dihydrolipoyl dehydrogenase | SLPGVTIDEK |

Table S6. Differentially expressed proteins involved in sucrose accumulation and fatty acid degradation in *O. longistaminata*.

| Gene Symbol | Enzyme Symbol | Protein Description | Ls8.5mm/Ls4mm | Ls8.5mm/Ls6mm |
| --- | --- | --- | --- | --- |
| **Glyoxylate and dicarboxylate metabolism** | | | | |
| BGIOSGA009926 | PCP | Glutamine synthetase [EC:6.3.1.2] | 6.18 | 1.18 |
| BGIOSGA023784 | GAT | Glutamine amidotransferase [EC:1.4.7.1] | 2.29 | 2.26 |
| BGIOSGA005667 | PCP3 | Glutamine synthetase [EC:6.3.1.2] | 6.41 | 5.34 |
| BGIOSGA011196 | PCP2 | Glutamine synthetase [EC:6.3.1.2] | 10.00 | 4.84 |
| BGIOSGA009738 | MDH | Malate dehydrogenase [EC:1.1.1.37] | 13.47 | 6.98 |
| BGIOSGA013524 | SHMT | Serine hydroxymethyltransferase [EC:2.1.2.1] | 6.05 | 4.95 |
| BGIOSGA038154 | RUBISCO | Ribulose bisphosphate carboxylase [EC:4.1.1.39] | 30.15 | 14.70 |
| BGIOSGA002081 | HPPR | Hydroxyphenylpyruvate reductase [EC:1.1.1.81] | 4.27 | 3.35 |
| BGIOSGA013686 | FMN | hydroxy acid dehydrogenase [EC:1.1.3.15] | 49.59 | 24.04 |
| **Fatty acid metabolism** | | | | |
| BGIOSGA011650 | PPT | Palmitoyl protein thioesterase [EC:3.1.2.22] | 3.59 | 2.77 |
| BGIOSGA015217 | ACP9 | Stearoyl-[acyl-carrier-protein] 9 [EC:1.14.99.6] | 2.91 | 2.91 |
| BGIOSGA018301 | C4H | Cinnamate-4-hydroxylase [EC:1.14.13.11] | 10.26 | 3.8179 |
| BGIOSGA018867 | ABP1 | AMP-binding domain-containing protein | 0.64 | 0.46 |
| BGIOSGA019284 | ACBD | Acyl-CoA dh M domain-containing protein | 6.4901 | 1.5249 |
| BGIOSGA021280 | ACO1 | Acyl-coenzyme A oxidase 1 [EC1.3.3.6] | 2.84 | 2.27 |
| BGIOSGA002347 | ACO4 | Acyl-coenzyme A oxidase 4 [EC1.3.3.6] | 2.10 | 2.31 |
| BGIOSGA005359 | KCT1 | 3-ketoacyl-CoA thiolase 1 [EC:2.3.1.15] | 2.4876 | 1.6229 |
| BGIOSGA033076 | KCT2 | 3-ketoacyl-CoA thiolase 2 [EC:2.3.1.16] | 1.8099 | 1.437 |
| BGIOSGA021279 | ACO | Acyl-coenzyme A oxidase [EC:1.3.3.6] | 3.1791 | 2.6012 |
| BGIOSGA032771 | ACC | Acetyl-CoA carboxylase [EC 6.4.1.2] | 2.6316 | 0.6055 |
| BGIOSGA031709 | GL1 | Very-long-chain aldehyde decarbonylase [EC:4.1.99.5] | 1.8538 | 0.7371 |
| **Glycolysis / Gluconeogenesis** | | | | |
| BGIOSGA011083 | PEP | Phosphoenolpyruvate carboxykinase [EC 4.1.1.32] | 4.8198 | 11.5346 |
| BGIOSGA010570 | SUS | Sucrose synthase [EC 2.4.1.13] | 1.3916 | 1.1866 |
| BGIOSGA008865 | PRK | Phosphoribulokinase [EC 2.7.1.19] | 28.1909 | 10.1126 |
| BGIOSGA000792 | PEP1 | Phosphoenolpyruvate carboxylase [EC 4.1.1.31] | 1.045 | 0.9323 |
| BGIOSGA026849 | GAD | Glutamate decarboxylase [EC:4.1.1.15] | 15.7175 | 7.5822 |

Table S7. Primer sequences used in qRT-PCR.

| Gene Symbol | Enzyme Symbol | Sequence (5'-3') | Product Length (bp) |
| --- | --- | --- | --- |
| BGIOSGA009926-PA | PCP | F:ATTGCTGGCGTCGTCGTGT | 296 |
|  |  | R:TCGTCCTTCTCGGTGTCCC |  |
| BGIOSGA011083-PA | PEP | F:AAGGGGTCGTTCATCACGTC | 95 |
|  |  | R:CATCCTTGACGATGCGCTTG |  |
| BGIOSGA015217-PA | ACP9 | F:CCTGGGCTGTTTGGACAAGA | 240 |
|  |  | R:TGGCGTGCCTTGCAGTATTA |  |
| BGIOSGA018301-PA | C4H | F:GAGCAGACTGGTGAGATCCG | 145 |
|  |  | R:TTCCCCATTCGATCGACCAC |  |
| BGIOSGA018867-PA | ABP1 | F:GTCAAAGCAAGGCTTGGTGG | 231 |
|  |  | R:AGGCACTTCTTCCAATCGCA |  |
| BGIOSGA031709-PA | GL1 | F:TCGACATGGACGAGTACCCT | 155 |
|  |  | R:TGCCAGTCCTGCAAGCATAA |  |
| BGIOSGA026849-PA | GAD | F:GACCAGATCATCCTGAGCGG | 263 |
|  |  | R:TAGGCAACGAGCTCAGCAAA |  |
| Os03g0718100 | Actin | F:TTCGGACCCAAGAATGCTAAG | 119 |
|  |  | R:AACAGATAGGCCGGTTGAAAAC |  |

Table S8. Peptide sequences of seven candidate proteins in PRM.

| Gene Symbol | Peptide Sequence | Retention Time | Ls6mm/Ls4mm  Ratio | Ls8.5mm/Ls4mm  Ratio | Ls8.5mm/Ls6mm  Ratio |
| --- | --- | --- | --- | --- | --- |
| PCP | WNFDGSSTGQATGDDSEVILHPQAIFR | 26.00 | 10.66 | 5.43 | 0.51 |
| PEP | VFVNDQFLNWDTENR | 19.40 | 0.62 | 3.09 | 5.01 |
| ACP9 | DDNLFEHFSAVAQR | 17.71 | 0.79 | 2.30 | 2.90 |
| C4H | FVPFGVGR | 15.67 | 2.78 | 10.41 | 3.74 |
| ABP1 | GVILDPIPFDLER | 22.89 | 1.26 | 0.51 | 0.40 |
| GL1 | VWLIGDGLDSAEQFR | 21.02 | 2.40 | 23.45 | 9.78 |
| GAD | LLNNLLTEK | 15.53 | 2.33 | 1.48 | 0.64 |
| GAT | VFTDEGLEVLGWR | 27.28 | 1.01 | 1.56 | 1.54 |
| PCP3 | WNYDGSSTGQAPGEDSEVILYPQAIFK | 27.72 | 1.19 | 5.77 | 4.83 |
| PCP2 | IIAEYIWVGGSGIDLR | 27.81 | 2.00 | 7.29 | 3.64 |
| MDH | VAVLGAAGGIGQPLSLLMK | 28.77 | 1.59 | 6.95 | 4.37 |
| SHMT | VADFFDAAVNLALK | 29.09 | 1.67 | 6.29 | 3.77 |
| RUBISCO | FETLSYLPPLTVEDLLK | 29.35 | 2.09 | 21.88 | 10.46 |
| HPPR | FGITTSQICMVGDR | 21.64 | 1.04 | 0.81 | 0.78 |
| FMN | VPYVIFCAPPSR | 23.39 | 1.55 | 3.04 | 1.96 |
| PPT | ETAWFGYYPDGGFDPVLPPQK | 27.92 | 1.55 | 3.04 | 1.96 |
| ACBD | VFEFFLSRPDLQTPVEMSTAAHR | 26.23 | 2.29 | 2.90 | 1.27 |
| ACO1 | IFSDAIEALR | 22.47 | 0.81 | 6.29 | 7.77 |
| ACO4 | GVILCIENALADAGVAK | 28.08 | 1.13 | 1.72 | 1.51 |
| KCT1 | DTYPEDLLTVVLK | 29.32 | 1.73 | 2.08 | 1.20 |
| KCT2 | EIVVSADDGIRPGTSLAVLSK | 21.9 | 1.18 | 1.47 | 1.25 |
| ACO | GWLTEPGPDAELR | 21.3 | 1.38 | 1.97 | 1.43 |
| ACC | SSSVVTDYVGYLSK | 22.49 | 3.67 | 1.87 | 0.51 |
| PRK | NFNPVYLFDEGSSITWVPCGR | 29.23 | 2.48 | 22.13 | 8.93 |
| PEP1 | LADLEAAPAALAR | 19.99 | 1.38 | 1.08 | 0.79 |
| SUS | GAVEACFGQCYDGCK | 14.71 | 0.94 | 115.32 | 122.88 |

Table S9. Peptide sequences of seven candidate proteins in PRM.

| Gene Symbol | Ls4mm | Ls6mm | Ls8.5mm | Gene annotation |
| --- | --- | --- | --- | --- |
| BGIOSGA009631-PA | 0.872 | 0.220 | -1.092 | - |
| BGIOSGA035393-PA | 0.772 | 0.358 | -1.130 | Xyloglucan endotransglucosylase/hydrolase  [Source:UniProtKB/TrEMBL;Acc:A2ZF20] |
| BGIOSGA028246-PA | -0.396 | 1.137 | -0.742 | Xyloglucan endotransglucosylase/hydrolase [Source:UniProtKB/TrEMBL;Acc:A2YSQ3] |
| BGIOSGA004154-PA | 0.140 | 0.923 | -1.063 | PF00150:Cellulase (glycosyl hydrolase family 5) |
| BGIOSGA029899-PA | 0.923 | 0.139 | -1.062 | - |
| BGIOSGA005432-PA | 1.065 | -0.146 | -0.919 | DNA replication licensing factor MCM5 [Source:UniProtKB/Swiss-Prot;Acc:B8AEH3] |
| BGIOSGA029493-PA | -0.057 | 1.027 | -0.970 | - |
| BGIOSGA000898-PA | 0.260 | 0.844 | -1.104 | - |
| BGIOSGA012830-PA | -0.060 | 1.029 | -0.969 | - |
| BGIOSGA028985-PA | 0.926 | 0.135 | -1.061 | PF00069:Protein kinase domain |
| BGIOSGA018610-PA | 0.660 | 0.491 | -1.151 | - |
| BGIOSGA013850-PA | 0.350 | 0.778 | -1.128 | - |
| BGIOSGA002017-PA | 0.859 | 0.238 | -1.09764 | PF07762:Protein of unknown function (DUF1618) |
| BGIOSGA005634-PA | -0.253 | 1.102 | -0.849 | PF04969:CS domain |
| BGIOSGA029208-PA | 0.593 | 0.561 | -1.155 | - |
| BGIOSGA021729-PA | 0.117 | 0.936 | -1.053 | PF08263:Leucine rich repeat N-terminal domain |
| BGIOSGA021700-PA | 0.679 | 0.470 | -1.148 | - |
| BGIOSGA024623-PA | 0.363 | 0.768 | -1.131 | Cellulose synthase  [Source:UniProtKB/TrEMBL;Acc:A2YJC4] |
| BGIOSGA016732-PA | 0.474 | 0.675 | -1.149 | PF00657:GDSL-like Lipase/Acylhydrolase |
| BGIOSGA002744-PA | 0.811 | 0.307 | -1.117 | PF14541:Xylanase inhibitor C-terminal|PF14543:Xylanase inhibitor N-terminal |

Table S10. Peptide sequences of seven candidate proteins in PRM.

| Gene Symbol | Ls4mm | Ls6mm | Ls8.5mm | Gene annotation |
| --- | --- | --- | --- | --- |
| BGIOSGA035511-PA | 0 | 0.001 | 1.999 | PF14368:Probable lipid transfer |
| BGIOSGA000008-PA | 0.003 | 0.002 | 2.995 | PF00190:Cupin |
| BGIOSGA006938-PA | 0.001 | 0.004 | 2.995 | - |
| BGIOSGA032752-PA | 0.002 | 0.004 | 2.994 | - |
| BGIOSGA019733-PA | 0 | 0.002 | 1.333 | - |
| BGIOSGA012258-PA | 0.002 | 0.004 | 1.997 | - |
| BGIOSGA016918-PA | 0.016 | 0.005 | 1.992 | - |
| BGIOSGA020837-PA | 0.001 | 0.005 | 1.665 | Beta-galactosidase [Source:UniProtKB/TrEMBL;Acc:A2YF97] |
| BGIOSGA037299-PA | 0.009 | 0.007 | 2.322 | - |
| BGIOSGA035761-PA | 0.013 | 0.014 | 2.972 | Purple acid phosphatase [Source:UniProtKB/TrEMBL;Acc:B8BN70] |
| BGIOSGA015005-PA | 0.009 | 0.016 | 2.317 | Glutamate decarboxylase [Source:UniProtKB/TrEMBL;Acc:A2XTZ1] |
| BGIOSGA014212-PA | 0 | 0.010 | 1.330 | PF00657:GDSL-like Lipase/Acylhydrolase |
| BGIOSGA020893-PA | 0.025 | 0.022 | 2.301 | - |
| BGIOSGA009455-PA | 0.025 | 0.025 | 2.625 | - |
| BGIOSGA017778-PA | 0.057 | 0.027 | 2.592 | PF03321:GH3 auxin-responsive promoter |
| BGIOSGA020552-PA | 0.044 | 0.028 | 2.604 | PF02458:Transferase family |
| BGIOSGA016363-PA | 0.010 | 0.022 | 1.986 | Beta-fructofuranosidase, insoluble isoenzyme 3 [Source:UniProtKB/Swiss-Prot;Acc:Q01IS8] |
| BGIOSGA010778-PA | 0 | 0.019 | 1.654 | PF00202:Aminotransferase class-III |
| BGIOSGA013767-PA | 0 | 0.023 | 1.978 | - |
| BGIOSGA028722-PA | 0.004 | 0.034 | 2.961 | PF00445:Ribonuclease T2 family |
